# Volume and Connectivity Differences in Brain Networks Associated with Cognitive Constructs of Binge Eating

**DOI:** 10.1523/ENEURO.0080-21.2021

**Published:** 2022-02-14

**Authors:** Bart Hartogsveld, Conny W. E. M. Quaedflieg, Peter van Ruitenbeek, Tom Smeets

**Affiliations:** 1 Department of Clinical Psychological Science, Faculty of Psychology and Neuroscience, Maastricht University, Maastricht MD 6200, The Netherlands; 2 Department of Neuropsychology and Psychopharmacology, Faculty of Psychology and Neuroscience, Maastricht University, Maastricht MD 6200, The Netherlands; 3 Center of Research on Psychological disorders and Somatic diseases (CoRPS), Department of Medical and Clinical Psychology, Tilburg School of Social and Behavioral Sciences, Tilburg University, Tilburg LE 5000, The Netherlands

**Keywords:** binge eating disorder, bulimia nervosa, cognitive control, negative affect, reward sensitivity, stress

## Abstract

Bulimia nervosa (BN) and binge eating disorder (BED) are characterized by episodes of eating large amounts of food while experiencing a loss of control. Recent studies suggest that the underlying causes of BN/BED consist of a complex system of environmental cues, atypical processing of food stimuli, altered behavioral responding, and structural/functional brain differences compared with healthy controls (HC). In this narrative review, we provide an integrative account of the brain networks associated with the three cognitive constructs most integral to BN and BED, namely increased reward sensitivity, decreased cognitive control, and altered negative affect and stress responding. We show altered activity in BED/BN within several brain networks, specifically in the striatum, insula, prefrontal cortex (PFC) and orbitofrontal cortex (OFC), and cingulate gyrus. Numerous key nodes in these networks also differ in volume and connectivity compared with HC. We provide suggestions for how this integration may guide future research into these brain networks and cognitive constructs.

## Significance Statement

Binge eating disorder (BED) and bulimia nervosa (BN) are common eating disorders and remain a major problem because of the association with a variety of health risks. This review shows that three cognitive constructs that underlie these disorders, namely reward sensitivity, cognitive control, and negative affect, can be explained in terms of brain activity differences in key brain networks. These activity differences are interpreted in light of differences in brain volume and connectivity, observed in different studies. Furthermore, the role of these networks involving the striatum, insula, prefrontal cortex (PFC) and orbitofrontal cortex (OFC), and cingulate gyrus, are interpreted by means of the current understanding of their function and mechanisms. Finally, suggestions for further research integrating brain function and structure in binge eating are made.

## Introduction

Despite widespread attention from the general public and the scientific community, bulimia nervosa (BN) and binge eating disorder (BED) remain a major societal problem, with an estimated prevalence of 2–3% (Stice et al., 2013). Because of unhealthy eating behavior and a strong association with obesity, these disorders are associated with a number of diseases, such as type II diabetes, cancer, cardiovascular diseases, and higher mortality rates (Hudson et al., 2007; Guh et al., 2009; Smink et al., 2012; Kessler et al., 2013; Patel et al., 2018). Both BN and BED are characterized by binge eating episodes, which have to meet two requirements (American Psychiatric Association, 2013). First, a certain amount of food has to be consumed within a 2-h period that is definitely larger than what most individuals would eat in a similar time period under similar circumstances. Second, the person has to experience a feeling that they cannot stop eating or control what or how much they are eating during the binge eating episode. Both BED and BN diagnosis require a frequency of these episodes of once a week on average, lasting for at least three months. In addition, stress regarding binging is a criterion for the diagnosis of both BN and BED (American Psychiatric Association, 2013). The disorders are also associated with several changes in eating patterns, such as eating much more rapidly than normal. However, only BN is accompanied by inappropriate compensatory behaviors to prevent weight gain, such as fasting, laxative use, or excessive exercise (American Psychiatric Association, 2013).

Given the numerous health risks, research into the mechanics of BN and BED is essential to improve our understanding of these disorders. Recent studies suggest that the underlying causes consist of a complex system of environmental cues, altered processing of food stimuli and behavioral responding, and brain differences. In particular, the literature indicates that three major cognitive constructs play a large role in BN and BED, namely reward sensitivity, cognitive control, and negative affect (for an overview, see Appelhans, 2009; Vainik et al., 2019). Reward sensitivity includes alterations in cue reactivity and hypo-responsiveness to food consumption (in which craving toward food is heightened but food liking when consumed is reduced), and serves as a motivational basis for impulsive behavior and engaging in binge eating (Giel et al., 2017). Cognitive control is highly connected to instrumental behavior and goal-directed behavior in particular, and reduced cognitive control may form the basis of losing control over ones eating behavior (Ridderinkhof et al., 2004; O’Doherty et al., 2017; Voon et al., 2017; Kar et al., 2019; Quaedflieg et al., 2019). Negative affect, or increased sensitivity to negative emotions (Vainik et al., 2019), is shown to be important in the development of BN and BED (Grilo and Masheb, 2002; Rojo et al., 2006; Allison et al., 2007; Striegel-Moore et al., 2007) and the frequency of binge eating episodes (Haedt-Matt and Keel, 2011; Berner et al., 2017a). Particularly, an increase in negative affect is shown to be strongly connected to and typically caused by stress (Mendonça-de-Souza et al., 2007; Wemm and Wulfert, 2017). Stress-induced changes in appetite related hormones and reward brain circuitry are an important factor in BN and BED (Gluck, 2006; Wierenga et al., 2018; for animal model, see Hardaway et al., 2015). Specifically, in BN and BED several physical differences in the stress response can be observed, such as hypothalamic-pituitary-adrenal (HPA) axis hyperactivity, elevated cortisol awakening response, and blunted responses to acute stressors compared with healthy controls (HC; Culbert et al., 2016; Wierenga et al., 2018; Naishe et al., 2019). We will demonstrate that functional imaging studies show differences in BN/BED specifically in brain areas associated with reward sensitivity, cognitive control, and negative affect as cognitive constructs underlying the disorders and that these overlap with gray matter (GM) volume and structural and functional connectivity differences.

To our knowledge, no review has integrated the findings of the three major cognitive constructs underlying BN and BED with structural and task-based neuroimaging studies in these disorders. The current narrative review is intended to supply an integrative account to identify key brain networks that are affected to aid further research. To do so, we will discuss (1) the differences between BN/BED and HC in brain networks associated with the three aforementioned cognitive constructs; and (2) differences in brain organization in BN/BED, including differences in GM volume and connectivity. Specifically, we will first give an overview of prominent studies related to the three cognitive constructs in BN/BED (e.g., food tasting, instrumental learning tasks, acute stress exposure). We will explore behavioral differences, their relation to physical measurements (e.g., cortisol response), and the connection to task-based brain activation studies. Subsequently, we will give an overview of differences in GM and white matter (WM) volume compared with HC, structural connectivity (diffusion weighted imaging studies) and functional connectivity (resting state studies). In the last section, we will integrate task-independent and task-dependent brain differences, discuss the potential function of these areas, and give suggestions for future research. As will be shown in this review, neuroimaging studies can help in understanding the neurobiological underpinnings of BN and BED, and so may aid in early detection or serve as a potential biomarker (Val-Laillet et al., 2015).

## Cognitive Constructs Associated with Binging Behavior

BEDs are, as previously mentioned, associated with differences in three major cognitive constructs (Vainik et al., 2019): reward sensitivity (Reward sensitivity), cognitive control (Cognitive control), and negative affect (Concepts and behavioral studies and Functional imaging studies). In the sections below, these three cognitive constructs are discussed, first in the context of binging behavior and subsequently in a neural context.

### Reward sensitivity

#### Concepts and behavioral studies

Reward sensitivity is the propensity to seek out rewards and encompasses both the anticipation of the reward and the actual affective experience during receiving the reward (Loxton and Tipman, 2017; Vainik et al., 2019). High reward-sensitive individuals are thought to more often seek out high palatable foods (Nansel et al., 2016); and thus, reward sensitivity may have similar behavioral effects in BN/BED. The two aspects of reward anticipation and the affective experience are represented in the distinction between “wanting” (i.e., anticipation) and “liking” (i.e., during receiving the reward; Robinson and Berridge, 1993). Berridge (1996) proposed that this distinction could affect the processing of food rewards, represented in two separate brain networks, although others have argued that this distinction cannot be easily made (Rogers and Hardman, 2015; Rogers, 2017). BN/BED could be explained by an increase in initial reward anticipation (i.e., wanting or craving), and repeated ingestion of that food could diminish its rewarding effects (i.e., liking; Berridge, 1996).

Changes in wanting and liking operates through Pavlovian conditioning, where food-related environmental cues (e.g., seeing a bag of crisps), elicit a learned response (i.e., wanting/anticipation). This response seems to be stronger in BN and BED compared with HC (Meule et al., 2018). Several aspects of the learning process can be affected in BN/BED, such as enhanced learning of stimuli-response associations, responses generalizing more easily to novel food stimuli, or an increased difficulty to extinguish craving (wanting; for an overview, see van den Akker et al., 2018).

Not only Pavlovian conditioning but also instrumental conditioning is likely to play a large role in BN/BED, operating mainly through behavioral responses (e.g., binge eating). In instrumental conditioning, responses to stimuli that lead to positive outcomes are reinforced, thereby increasing the probability of that response being repeated. In the literature this association between a stimulus, response (e.g., pressing a button), and outcome (e.g., a food reward) is often abbreviated to S-R-O. If this sequence is repeated several times, the outcome (O) is thought to lose its functional role and the stimulus will thus automatically come to elicit the response (S-R). In the example, a food reward is no longer necessary to elicit the response (R), the stimulus (S) is sufficient to generate a response, leading to a S-R association (for a complete overview, see O’Doherty et al., 2017). These association-based behaviors are also known as goal-directed (S-R-O) and habitual behavior (S-R), respectively, and are related to differences in cognitive control in BN/BED, the second important cognitive construct (Vainik et al., 2019). Patients with BED appear to rely more often on habitual behavior compared with HC (Voon et al., 2015). Thus, the insensitivity to reward value (i.e., outcome) in habitual behavior may explain the behavioral hypo-responsiveness for the reward value experienced during binge eating episodes, although large quantities of food are consumed (Berner et al., 2017b). Most studies that investigated reward sensitivity experimentally suggest higher reward sensitivity on a subjective level and in food-related tasks (Schag et al., 2013; Loxton and Tipman, 2017; for systemic review, see Harrison et al., 2010). However, this effect does not correspond well with studies using non-food-related tasks where often no differences between BN/BED and HC are found (Appelhans, 2009; Kessler et al., 2016; Simon et al., 2016; Giel et al., 2017; Rogers, 2017; Hutson et al., 2018; Wierenga et al., 2018; Balodis et al., 2019). From the behavioral results it seems clear that reward sensitivity is not consistently higher in BN/BED across all reward types and that it might be limited to food rewards alone (Appelhans, 2009)

#### Functional imaging studies

**Figure 1.**
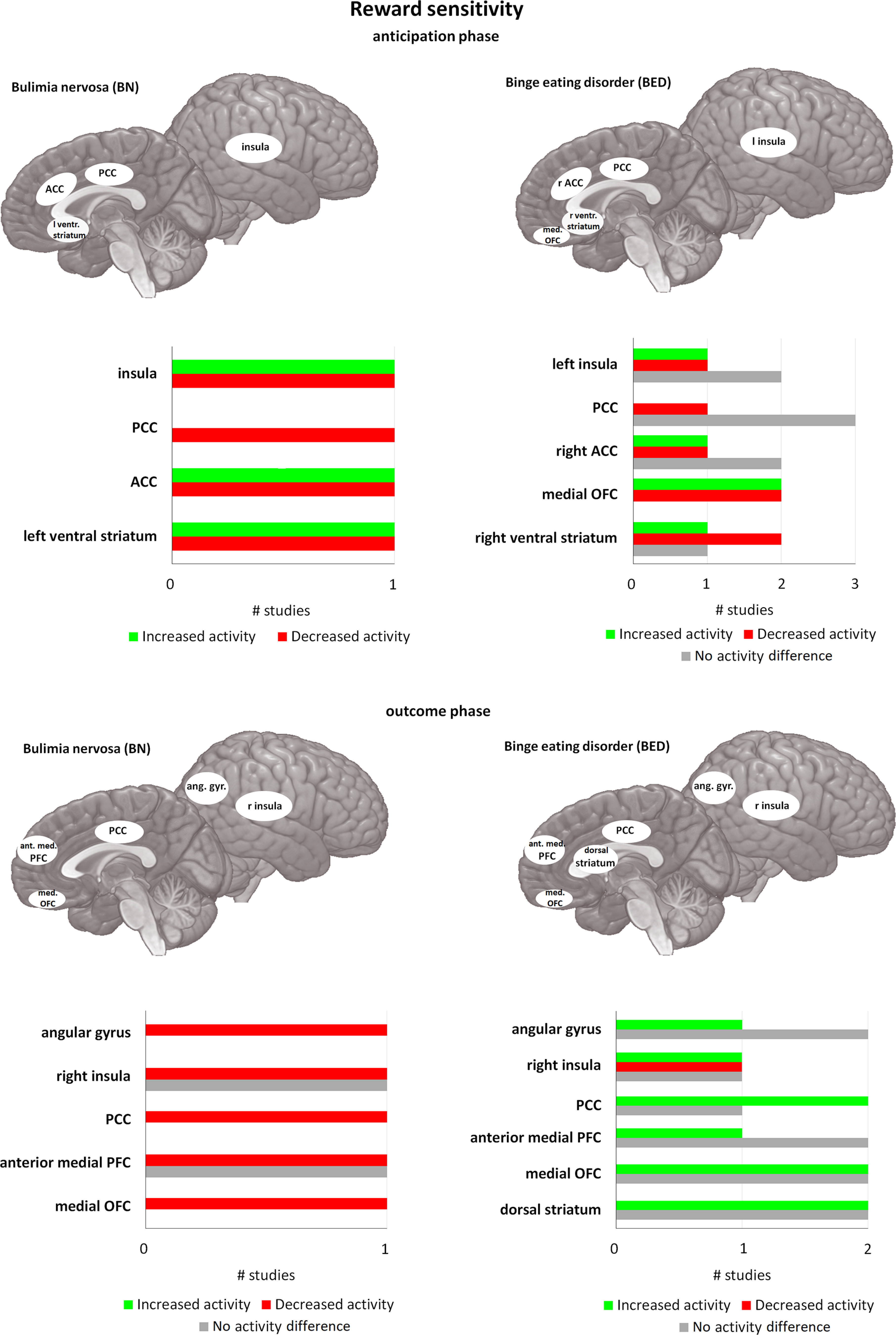
Differences in activity in BN and BED in passive food viewing tasks and active food tasting tasks compared with matched controls. For each area, the bar graph indicates the number of studies that found a decrease in activity (= red), or an increase in activity (= green), and the studies that found no difference in activity (= gray), during passive food viewing tasks (i.e., studies using paradigms where participants had to merely view food stimuli; shown in the two images on top) and active food tasting tasks (i.e., studies where participants had to actively taste food in the scanner; represented in the two images at the bottom). All of the included studies found at least one brain area that was significantly different in activation in BN and/or BED compared with HC. PCC = posterior cingulate cortex, ACC = anterior cingulate cortex, ventr. striatum = ventral striatum, med. OFC = medial orbitofrontal cortex, ant. med. PFC = anterior medial prefrontal cortex, ang. gyr. angular gyrus. If no indication of lateralization is given (either left or right), differences are observed bilaterally.

Using a variety of reward sensitivity tasks, several differences in brain activation are observed between BN and BED and HC (see Table 1; Fig. 1). Brain areas where differences in activation were found were the anterior cingulate cortex (ACC) and posterior cingulate cortex (PCC), the ventral striatum, and the insula, all of which are involved in reward sensitivity (Schienle et al., 2009; Weygandt et al., 2012; Balodis et al., 2013a, 2014; Oberndorfer et al., 2013; Simon et al., 2016). The ACC has been associated with reward learning and outcome prediction (Alexander and Brown, 2019), and the PCC with storing and retrieving reward values (Rolls, 2019). The ventral striatum is thought to compare these predicted reward values with the outcome (Wang et al., 2016). Finally, the insula is typically implicated in the actual somatosensory experiences such as taste and touch (Rolls, 2016).

**Table 1:** Overview of publications on reward sensitivity, cognitive control, and negative affect in BN and BED

Study	Subjects	Gender (♀/♂)	Age $\bar{\text{x}}$ (SD)	BMI $\bar{\text{x}}$ (SD)	Diagnosis	Task	Method	Findings
Reward sensitivity								
BN								
Oberndorfer et al. (2013)***^f^***	BN recovered (14)	14/0	26.6 (5.7)	22.9 (2.1)	SCID DSM-IV-TR	Taste of sucrose and sucralose	MRI, whole brain, ROI	Increased activation right AI during sucrose in BN compared with HC
	HC (14)	14/0	27.4 (5.5)	22.6 (1.5)				
BED								
Filbey et al. (2012)*^g^*	BED-spectrum (26)	14/12	32.88 (11.04)	32.72 (5.98)	BES > 8	Food cue exposure task	MRI, whole brain; PPI	Taste cues high-caloric food vs neutral mOFC, VTA, NAc, insula, caudate, putamen, precuneus, CG, thalamus, amygdala, hippocampus
								Positive correlation compulsive overeating scores with activity in: amygdala, putamen, insula, PCC, precuneus, hippocampus, thalamus, CG, VTA, MFG
								Functional connectivity during high-caloric cues vs neutral NAc more positively correlated with mOFC and DS NAc connectivity with PCC/precuneus positively correlated with BES scores
Balodis et al. (2013a)***^f^***	BED (19)	14/5	43.7 (12.7)	36.7 (4.05)	DSM-5	Monetary Incentive Delay task	MRI, whole brain	HC,OB>BED, decreased activity VS (anticipation of reward) PFC and insula (outcome phase)
	OB (19)	10/9	38.3 (7.5)	34.6 (3.5)				
	HC (19)	10/9	34.8 (10.7)	23.3 (1.1)				
Balodis et al. (2014)***^f^***	Remain BED (10)	14/5, difference N.S.	43.7 (12.7) difference, N.S.	36.7 (4.05), difference N.S.	DSM-5, re-analysis of data (Balodis et al., 2013)	Monetary Incentive Delay task	MRI, whole brain	Remain BED group diminished VS and IFG in anticipation, reduced activity in medial PFC during outcome phase
	Recovered BED (9)	14/5, difference N.S.	43.7 (12.7), difference N.S.	36.7 (4.05), difference N.S.	Recovered is no binge eating last 28 d			
Wang et al. (2011)***^g^***	BED (10)	8/2	38.5 (13.3)	43.4 (13.5)	DSM-IV, EDE	Food cue and taste task (smell/view/taste); administered methylphenidate (MPH)	PET, [11C]raclopride	N.S., in placebo Food stimuli in MPH condition increased dopamine in caudate and putamen in BED, not in OB Dopamine increases caudate positively correlated binge eating scores, not BMI
	OB (8)	5/3	41.8 (8.9)	36.5 (9.4)				
BN and BED								
Schienle et al. (2009)***^g^***	BED (17)	17/0	26.4 (6.4)	32.2 (4)	DSM-IV, EDI	Passive food viewing, food/disgust/neutral pictures	MRI, whole brain/ROI	Between-subject, food>neutral BED>OB, mOFC BED>HC, right mOFC BED>BN, right lOFC, right mOFC BN>OB, ACC, right insula BN>HC, right ACC, insula BN>BED, ACC, right insula
	BN (14)	14/0	23.1 (3.8)	22.1 (2.5)				Within-subject, food>neutral Occipital cortex, OFC, ACC, insula
	OB (17)	17/0	25 (4.7)	31.6 (4.7)				Reward sensitivity scores positively correlated with activity to food>neutral In BED: left ACC, right mOFC In BN: insula, ACC, mOFC
	HC (19)	19/0	22.3 (2.6)	21.7 (1.4)				Arousal scores positively correlated with activity to food>neutral In BED: ACC, mOFC
								EDI binge eating scores positively correlated with activity to food>neutral In BN: right insula
Simon et al. (2016)***^g^***	BED (27)	N.R.	38.26 (13.75)	32.61 (4.55)	SCID DSM-5	Monetary and Food Delay task	MRI, ROI	Anticipation of reward (high vs no-food reward) ROI: N.S. in striatum Whole brain: decreased PCC, HC>BN and HC>BED
	Controls BED (28)	Matched	38 (10.85)	34.02 (4.5)				Receipt of reward (high vs no-food reward) ROI: increased mOFC, BN>HC and BED>HC Whole brain: increased PCC, anterior medial PFC, AG, BN>HC and BED>HC
	BN (29)	N.R.	27.45 (10.55)	21.33 (2.99)				N.S. in monetary delay task
	Controls BN (27)	Matched	25.74 (5.25)	21.85 (1.85)				
	BN (14)	14/0	23.1 (3.8)	22.1 (2.5)				Activity predicts group for BED and HC: right insula, left lOFC BED and OB: right ACC, left insula, mOFC, right VS BN and HC: left ACC, right insula, left VS BN and OB: right lOFC BED and BN: right ACC, insula, left VS
	OB (17)	17/0	25.0 (4.7)	31.6 (4.7)				
	HC (19)	19/0	22.3 (2.6)	21.7 (1.4)				
Cognitive control								
BN								
Marsh et al. (2011)***^g^***	BN and BN-spectrum (18)	18/0	18.4 (2.1)	22.03 (2)	N.R., 4 subclinical	Simon task	MRI, whole brain	Greater deactivations in BN compared with HC in: left IFG, left SFG, right MFG, right PCC
	HC (18)	18/0	17.3 (2.4)	21.98 (1.9)				Greater activations in HC compared with BN in right: putamen, hippocampus, IFG, ACC, dlPFC Objective bulimic and vomiting episodes and preoccupation with weight scores correlated in BN with: Negatively: left IFG, precuneus, right putamen Positively: left SFG, left insula, right ACC
Neveu et al. (2018)***^g^***	BN (31)	31/0	24 (3.87)	19.9 (2.5)	Patients, DSM-IV	Food evaluation choice task	MRI, whole brain	Correlation between vmPFC activity and health rating was more negative in BN than HC In BN and HC, positive correlation between uncontrolled choices and activity dlPFC In BN and HC, positive correlation between uncontrolled choices and connectivity dlPFC-vmPFC
	HC (23)	23/0	23 (2.7)	21.3 (2.36)				
BED								
Balodis et al. (2013b)***^g^***	BED (11)	9/2	47.6 (12.7)	37.1 (3.9)	SCID DSM-5, EDE-Q	Stroop task	MRI, whole brain	All groups showed consistent Stroop-related differences in incongruent>congruent e.g., insula, cingulate, striatum, thalamus, frontal gyri, cerebellum, cuneus
	OB (13)	5/8	35.4 (9.3)	34.6 (4.1)				Group differences on incongruent>congruent OB>BED, decreased activity IFG, MFG, SFG, OFG, MOG, IOG, LG, STG, insula, PreCG HC>BED, decreased activity SOG, MOG, STG, insula
	HC (11)	5/6	32.7 (11.3)	23.2 (1.1)				
Hege et al. (2015)***^f^***	BED (13)	13/0	41.15 (9.14)	33.45 (5.38)	SCID, DSM-IV-TR, EDE-Q	Food-related visual Go/No-go	MEG	Successful withholds during no-go trials associated with SFG and right SMG BIS-11 negatively correlated with right MFG activity; BED showed decreased activity on food stimuli on successful withholds in no-go trials
	OB (16)	16/0	40.19 (11.73)	36.38 (5.02)				
Voon et al. (2015)***^g^***	BED (31)	19/12	42.79 (9.02)	34.97 (5.56)	DMS-IV-TR	Sequential instrumental learning task	MRI, VBM	In non-related HC group, bias toward goal-related responding positively correlated with volume in left mOFC, caudate, and lateral PFC In subsample, adding HAB/GD as covariate, previously described differences (see Table 2) disappeared
	OB (31)	12/19	44.24 (9.39)	31.49 (3.6)				
Negative affect								
BN								
Collins et al. (2017)***^g^***	Sample 1							HC>BN, decreased activity: precuneus, PaCG, anterior vermis
	BN (10)	10/0	21 (2.5)	21.75 (1.59)	DSM-5, SCID, EDE	Visual food cue processing task	Whole brain	BN prestress > BN poststress, decreased activity: precuneus, PaCG, anterior vermis
	HC (10)	10/0	24 (5.5)	22.21 (1.28)		TSST (only mathematical part)		
Collins et al. (2017)***^g^***	Sample 2							Replicated findings study 1
	BN and OSFED-BN (17)	17/0	22.85 (5.42)	24.47 (3.25)	DSM-5, SCID, EDE	Visual food cue processing task TSST (only mathematical part)	ROI	
Fischer et al. (2017)***^g^***	BN and BN-spectrum (16)	16/0	22.85 (5.42)	24.47 (3.25)	DSM-5, 12 BN, 4 OSFED and BN symptoms, EDE	Visual palatable food cue processing, TSST	WS, ROI	BN prestress > BN poststress, decreased activity: vmPFC, right ACC, left amygdala
Wonderlich et al. (2018)***^g^***	BN and BN-spectrum (16)	16/0	22.85 (5.42)	24.47 (3.25)	DSM-5, 12 BN, 4 OSFED and BN symptoms, EDE, re-analysis of data (Fischer et al., 2017)	Visual palatable food cue processing, TSST	EMA-fMRI integration, WS, ROI	Predictive of negative affect (high just before binge): right amygdala, vmPFC Predictive of positive affect (high before and after binge): left amygdala, right ACC, vmPFC
Lyu and Jackson (2016)***^g^***	BED, stress (9)	9/0	19.22 (0.44)	20.8 (1.48)	DSM-IV	Visual food cue processing (high/low-caloric, neutral images)	BS, ROI	Stress condition, HC>BED, decreased activity High-caloric vs neutral: IFG, insula, hippocampus
	BED, no-stress (9)	9/0	19.89 (1.54)	20.72 (2.34)		CPT		Low-caloric vs neutral: hippocampus
	HC, stress (12)	12/0	20 (1.41)	19.19 (1.52)				High-caloric vs low-caloric: IFG, hippocampus, amygdala
	HC, no-stress (14)	14/0	19.43 (1.34)	19.22 (2.16)				Control condition, HC>BED, decreased activity High-caloric vs neutral: SFG, ACC
								Low caloric vs neutral: SFG, ACC, putamen
								High-caloric vs low-caloric: PaCG

N.S. = not significant, N.R. = not reported.

Abbreviations subjects: BN = bulimia nervosa, BED = binge eating disorder, HC = healthy control, OB = obese control, OSFED = other specified feeding or eating disorders.

Abbreviations diagnosis: SCID = structured clinical interview for the DSM, DSM = diagnostic and statistical manual of mental disorders, DSM-IV-TR = DSM IV textual revision, EDE = eating disorder examination interview, EDE-Q = EDE questionnaire, EDI = eating disorder inventory, QWEP = questionnaire on eating and weight patterns, BES = binge eating scale.

Abbreviations task: TSST = Trier Social Stress Task, CPT = Cold Pressor Test.

Abbreviations method: VBM = voxel-based morphometry, ROI = region-of-interest analysis, PPI = psychophysiological interaction, WS = within-subject, BS = between=subject, EMA = Ecological Momentary Assessment.

Abbreviations findings general: HAB/GD = habitual/goal-directed responding.

Abbreviations findings areas: AI = anterior insula, VTA = ventral tegmental area, NAc = nucleus accumbens, DS = dorsal striatum, VS = ventral striatum.

Abbreviations findings cortex: mOFC = medial orbitofrontal cortex, lOFC = lateral OFC, vmPFC = ventromedial prefrontal cortex, dlPFC = dorsolateral PFC.

Abbreviations findings gyri: CG = cingulate gyrus, MFG = medial frontal gyrus, IFG = inferior frontal gyrus, SFG = superior frontal gyrus, OFG = orbitofrontal gyrus, PreCG = precentral gyrus, AG = angular gyrus, LG = lingual gyrus, MOG = middle occipital gyrus, IOG = inferior occipital gyrus, SOG = superior occipital gyrus, STG = superior temporal gyrus, SMG = superior medial gyrus, PaCG = paracingulate gyrus. *^g^* and *^f^* refer to the quality assessment done by two independent raters (for more details, see Concluding Remarks, Quality assessment). Represents a good (≥7.5) or a fair (4–7.5; out of 10) rating, respectively.

In more detail, in the insula and ACC, food cue exposure increases brain activation in BN, BED, and HC (Schienle et al., 2009; Wang et al., 2011). Yet in BED and particularly in BN, this increase appears larger compared with HC (Schienle et al., 2009; Weygandt et al., 2012; Oberndorfer et al., 2013). In contrast, activation of the PCC in BN and BED was decreased in an anticipatory task compared with HC, while it was increased in a consummatory task (Simon et al., 2016). Using a monetary incentive delay task, obese BED showed lower activity in ventral striatum during anticipation of reward, and in the prefrontal cortex (PFC) and insula in the outcome phase compared with obese non-BED and HC (Balodis et al., 2013a). The regional specific responses again point to differences in anticipating and receiving rewards. While the role of the cingulate cortex and insula are not domain-specific, the ventral striatum has a specific role in the reward network.

All in all, research shows clear differences in food-specific reward sensitivity in BN and BED. This is supported by the results of a study demonstrating that subjects could be successfully identified as BED, BN, HC, and obese with an accuracy of 59–90% based on activity patterns of the insula, ventral striatum, ACC, and the orbitofrontal cortex (OFC; Weygandt et al., 2012). In the future this could add further specificity to diagnosis of BN or BED. This seems to be partially associated with posttreatment outcome. BED patients who continued to report binge eating after treatment, were shown to have lower activity of ventral striatum and inferior frontal gyrus (IFG) during anticipation phase, and lower medial PFC activation during the outcome phase versus recovered patients (Balodis et al., 2014).

### Cognitive control

#### Concepts and behavioral studies

Cognitive control is multi-dimensional in that it covers various aspects of cognitions and behavior, such as planning, attention/distraction, motoric impulsivity, non-planning impulsivity or lack of concern for the future (Fischer et al., 2003; Rosval, 2006; Kessler et al., 2013; Fineberg et al., 2014). Not all of these cognitive components are unambiguously affected in BN/BED compared with HC. The most notable differences are found in impulsivity during food-related tasks (Fischer et al., 2003; Waxman, 2009; Wu et al., 2014). However, some authors (Neveu et al., 2018) have emphasized that BN/BED patients cannot be fully identified by their loss of control. Because binges can be planned in advance, BN subjects are able to postpone a binge if conditions such as food availability are not met, and subjects often choose food that is often consumed outside binges. Thus, in BN/BED loss of cognitive control is not a domain-general failure, but seems specific to the binging episodes (Neveu et al., 2018). However, in BED subjects planned binges are often longer and larger than initially intended by the subject (Curtis and Davis, 2014). This indicates that a deficit in cognitive control, resulting in a behavioral loss of control, was found to be primarily present during the binge itself (also see Adams et al., 2019). Crucial to both BN and BED is the specific, food-related loss of control during the binge eating episode itself, as illustrated by an increase in intake, even if the episode is planned beforehand (Curtis and Davis, 2014). This loss of control could be explained through a switch from goal-directed to habitual behavior, where direct S-R associations are able to drive behavior (Watson et al., 2014; Cartoni et al., 2016; Watson et al., 2017). Of course, it is not necessary that this loss of control always leads to an increase in intake, as seen in subjective binge eating (where there is only an experience of loss of control).

Since cognitive control is multi-dimensional it is measured by a variety of tasks and self-reported questionnaires [e.g., stop-signal task (SST), Stroop, Go/No-go, memory paradigms, Simon tasks, delay discounting; see Bartholdy et al., 2016; Lavagnino et al., 2016]. Using self-reported questionnaires, BED patients show increased impulsivity, elevated compulsivity, decreased self-control, impaired set-shifting reflective of compulsive behavior, motoric impulsivity, attention impulsivity, and a higher tendency to seek out novel experiences and stimuli (Fahy and Eisler, 1993; Claes et al., 2002; Favaro et al., 2005; Rosval, 2006; Galanti et al., 2007; Danner et al., 2012; Hege et al., 2015; Kessler et al., 2016). Self-reported cognitive control was positively associated with task performance or amount of food eaten during the experiment (Galanti et al., 2007; Hege et al., 2015; Kessler et al., 2016). Other studies, including two meta-analyses, confirm the important role of self-reported cognitive control, but also report that not all components are consistently found (Fischer et al., 2003; Waxman, 2009; Wu et al., 2014). In particular, while planning does not seem to be impaired, acting rashly, general impulsivity, and impairments in set-shifting do seem present in both BED and BN in these studies.

These diversely affected subcomponents of cognitive control further demonstrate that it is not a solitary cognitive construct. Evidence of cognitive control impairments in BN and BED obtained from experimental paradigms is equally inconsistent. Several systematic reviews and meta-analyses (Kittel et al., 2015; Lavagnino et al., 2016) demonstrate that differences in inhibitory responding in BN and BED are often task-dependent. Only around half of the studies included in these meta-analyses showed impaired performance on relevant tasks by patients compared with HC matched on BMI (body mass index; e.g., SST, Stroop task; Kittel et al., 2015; Bartholdy et al., 2016; Lavagnino et al., 2016). Results of the SST indicate that the observed impairments are because of reduced inhibitory control and not to motor response deficits. Overall SST scores correlate with eating pathology in BED but not in BN. Two other studies showed that the impairment in inhibitory responding (using a Simon and Go/No-go task) might be related to symptoms of BN (Bruce et al., 2003; Marsh et al., 2009). Symptom severity (e.g., number of binge eating and vomiting episodes) was inversely correlated with task accuracy and activation of the frontostriatal network (Marsh et al., 2009). A systematic review showed that inhibition and delay gratification deficits might actually be limited to food related tasks (Kittel et al., 2015). Similarly, Svaldi et al. (2014) found that inhibition was more impaired for food stimuli (however, see Manasse et al., 2016).

#### Functional imaging studies

**Figure 2.**
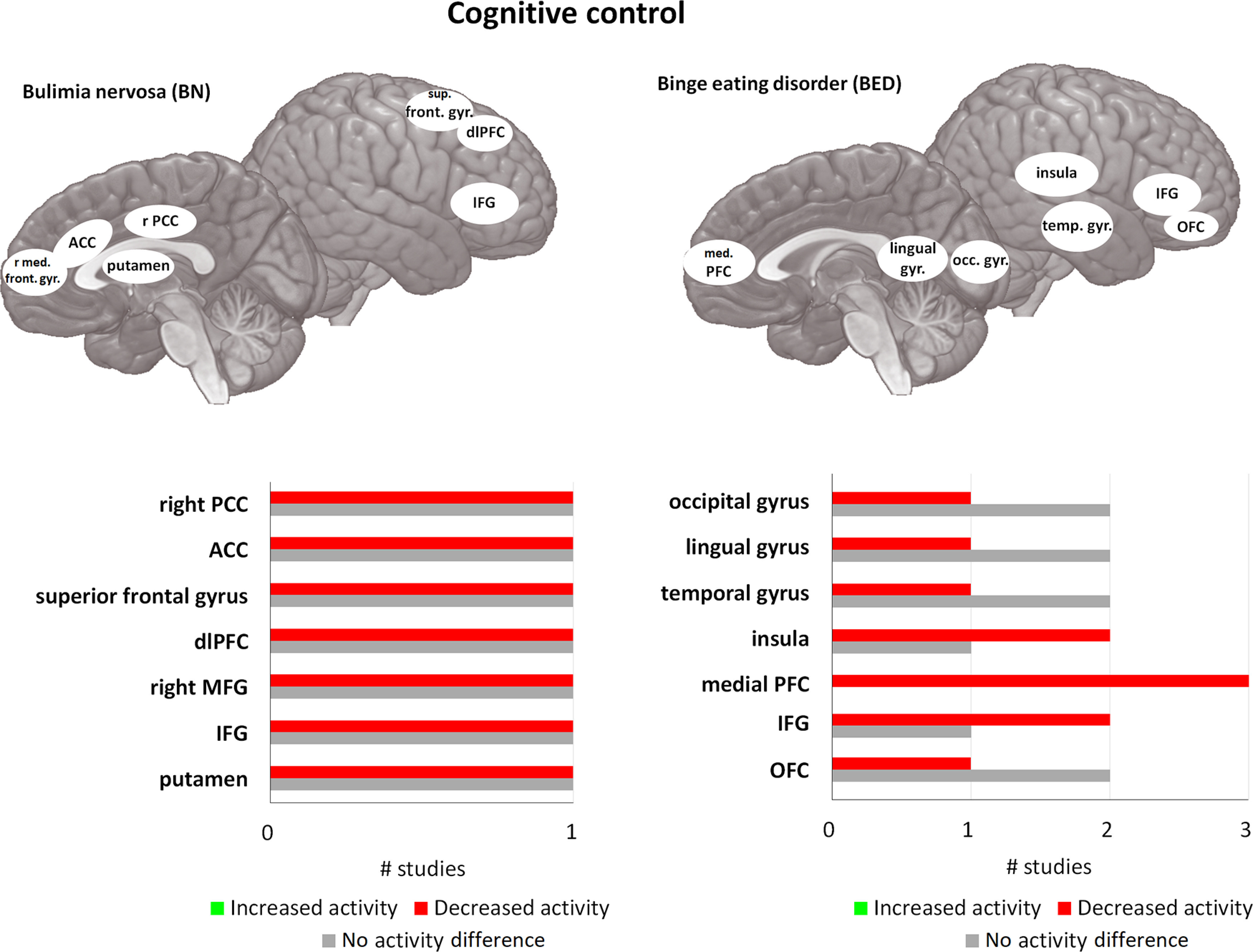
Differences in activity in BN and BED in cognitive control tasks compared with matched controls. For each area, the bar graph indicates the number of studies that found a decrease in activity (= red), or an increase in activity (= green), and the studies that found no difference in activity (= gray), during cognitive control tasks. All of the included studies found at least one brain area that was significantly different in activation in BN and/or BED compared with HC. med. front. gyr. = medial frontal gyrus, PCC = posterior cingulate cortex, ACC = anterior cingulate cortex, IFG = inferior frontal gyrus, sup. front. gyr. = superior frontal gyrus, dlPFC = dorsolateral prefrontal cortex, med. PFC = medial prefrontal cortex, temp. gyr. = temporal gyrus, lingual gyr. = lingual gyrus, occ. gyr. = occipital gyrus. If no indication of lateralization is given (either left or right), differences are observed bilaterally.

Compared with the inconsistent findings in self-reported and behavioral tasks assessing cognitive control, imaging studies do show a consistent reduction in task-related brain activity in the associated PFC network. As proposed by the schematic control theory (Badre and Nee, 2018), the frontal areas can be divided into three subnetworks. This network consists of anterior PFC areas involved in schematic control (organizing features and relations), rostral mid-lateral PFC areas involved in contextual control (implementing behavioral strategies), and caudal frontal areas involved in sensory-motor control (executing behavior and sensory feedback; Badre and Nee, 2018). The reduced task-related brain activity has been found in all three networks for both BN and BED compared with HC, using a variety of paradigms (tasks are for example the SST, Simon, and Stroop; see Table 1; Fig. 2). A meta-analysis (Lavagnino et al., 2016) reported two studies investigating BED subjects. In one, obese BED subjects showed lower activation of the dorsolateral PFC (dlPFC) and IFG compared with obese non-BED in a food-specific Go/No-go task (Hege et al., 2015). Obese BED subjects also scored higher on a self-reported impulsiveness measure, and in both obese BED and obese non-BED this score was negatively correlated with response inhibition and activity in the prefrontal networks. BED subjects showed reduced activity in OFC, IFG, superior temporal gyrus, ventromedial PFC (vmPFC), and insula compared with HC and obese subjects during a Stroop task (Balodis et al., 2013b), while no differences were found in task performance. In addition, BED showed the highest self-reported dietary restraint scores, and this was negatively correlated with activation in vmPFC, OFC, IFG, and insula. In obese subjects, however, these scores were positively correlated with IFG and insula activation in obese subjects (Balodis et al., 2013b). This shows that there is a strong relation between measures of cognitive control and activation patterns during a task involving cognitive control. During the Simon task, BN patients showed decreased activation compared with HC in the bilateral IFG, lenticular and caudate nuclei, and ACC when responding correctly to incongruent stimuli (Marsh et al., 2011). In short, these studies show an overall reduction in activity in cognitive control networks, particularly several frontal areas.

As mentioned before, instrumental conditioning is highly related to cognitive control. Indeed, some authors have proposed that instrumental conditioning is implicated in many of the tasks used to measure cognitive control (Liefooghe, and de Houwer, 2016). It is indeed possible that a shift from goal-directed to habitual control likely precedes or occurs during a binge eating episode. One functional magnetic resonance imaging (fMRI) study directly assessed this possibility in obese BED and obese non-BED (Voon et al., 2015). Using a two-step sequential learning task, Voon et al. (2015) showed that obese BED had a lower engagement of a model-based strategy compared with obese non-BED and lean HC. This demonstrates a shift away from goal-directed toward habitual responses. In BED, higher scores on a binge eating scale were negatively associated with the amount of goal-directed responding during the task. In other words, the more severe BED was, the more likely the person was to respond in a habitual fashion. A subsample of the BED subjects in the Voon et al. (2015) study showed reduced volume in the left ventral striatum, left lateral OFC, bilateral medial OFC, and bilateral caudate compared with non-BED obese. When adding the goal-directed/habitual score as a covariate, Voon et al. (2015) found that the differences in medial OFC, caudate, and ventral striatum disappeared, indicating that the structural differences likely drive the differences in habitual and goal-directed responding between these groups. This is likely to be associated with a reduction in activation of networks associated with goal-directed responding, rather than a simple increased activation in networks associated with habitual responding. Overall, then, habit formation in BED could very well be mediated by the medial OFC-caudate-striatal network. These findings are consistent with those in BN. A recent study by Neveu et al. (2018) did demonstrate the involvement of vmPFC and dlPFC in a cognitive conflict task in BN, where subjects had to choose between healthy and tasty food. The authors proposed that the results of this study demonstrated increased goal-directed responding in BN. However, it is important to note that vmPFC activity can also be illustrative of value estimation (Wunderlich et al., 2012), rather than goal-directed responding.

Taken together, while differences on a behavioral level are limited and inconsistent, information processing in the brain is consistently different in subjects with BN or BED. Both seem to be characterized by an overall hypoactivity in frontal regions (OFC, vmPFC, dlPFC, IFG), ventral striatum, and insula in a variety of tasks measuring cognitive control. Additionally, decreased activation in the caudate nucleus in the aforementioned Simon task (Marsh et al., 2011) corresponds well with the interpretation that behavior could be biased toward habitual and away from goal-directed control (Voon et al., 2015). This is particularly likely since the caudate nucleus is hypothesized to be part of the goal-directed network together with frontal areas such as OFC, and the insula (Watson et al., 2018). These fMRI paradigms indicate that while there may not always be a behavioral difference between BN, BED, obese, and HC, differences in activation are often present. This illustrates a difference in processing that together with increased reward sensitivity, plays a large role in eating behavior in BN and BED.

#### Concepts and behavioral studies

Negative affect refers to increased sensitivity to negative stimuli and increased likelihood to experience negative emotions (Vainik et al., 2019) and is also highly related to the balance between habitual and goal-directed behavior. Heightened negative affect is typically the result of stress (Mendonça-de-Souza et al., 2007; Wemm and Wulfert, 2017), alters this balance in instrumental learning tasks, rendering behavior more habitual (Schwabe and Wolf, 2009, 2010; Schwabe et al., 2012; Wirz et al., 2018; Quaedflieg et al., 2019; Smeets et al., 2019; Hartogsveld et al., 2020). Negative affect and stress are also closely related to the concept of “comfort eating,” the idea that some people have an increased tendency to consume food when stressed, to relieve stress and improve affect (Gibson, 2012). Whilst support is inconsistent, several studies demonstrate that stressed (HC) subjects show a clear preference for food high in sugar and fat (but see Bongers and Jansen, 2016). Some authors have therefore suggested that bulimic episodes could be a form of learned behavior through negative reinforcement (Berner et al., 2017b; Racine and Martin, 2017; Wierenga et al., 2018). One proposed model to explain this further is the acquired preparedness model of binge eating (Racine and Martin, 2017). This model poses that certain high-risk personality traits, such as impulsivity, can alter learning through reward value and make it more likely that food is seen as a positive stimulus that can temporarily alleviate negative affect. Indeed, it has been shown that impulsivity under stress is associated with a greater risk for dysregulated eating in people with BN symptoms (Fischer et al., 2018). This association is contingent on the belief that food can alleviate negative affect, body dissatisfaction, and dietary restraint (Racine and Martin, 2017). It also might be connected to overall self-reported difficulties in emotion regulation in BN/BED compared with HC (Troop et al., 1998; Brockmeyer et al., 2014). Lastly, sensitivity to negative effect has also been associated with a higher frequency of binge eating episodes, greater severity of the disorder (Carrard et al., 2012), and playing a role in the development of BN/BED (Razzoli et al., 2016). For example, a meta-analysis revealed that negative affect increased before binge eating episodes compared with diurnal affect and affect before normal meals (Haedt-Matt and Keel, 2011). After this increase, negative affect decreases following the binge eating episode (Johnson and Larson, 1982; Kaye et al., 1986; Crosby et al., 2009; Berner et al., 2017a).

Stress may also be considered a form of negative affect. In support of the role of stress in BN/BED are the multiple alterations in stress hormone levels that are observed in BN/BED. Stress exposure causes a rapid release of catecholamines (e.g., adrenalin and noradrenalin) and activates the slower-acting HPA axis that results in the release of glucocorticoids (e.g., cortisol) in the blood. Collectively, these fast and slow stress hormones alter the responding of numerous circuits in the brain and other aspects of homeostasis (Ulrich-Lai and Herman, 2009). BN and BED are associated with differences in these hormones and circuits, namely long-term HPA axis hyperactivity, elevated cortisol awakening response, ultimately resulting in a blunted response to acute stressors (Culbert et al., 2016; Wierenga et al., 2018; Naishe et al., 2019).

In a lab setting, several studies have experimentally investigated acute stress-induced changes in eating behavior in BED, since acute stress is relatively easily and reliably inducible (Laessle and Schulz, 2009; Schulz and Laessle, 2012; Lyu and Jackson, 2016; Klatzkin et al., 2018). Acute stress mostly seems to affect eating rate, with a faster initial eating rate and smaller decrease in eating rate over time in BED (Laessle and Schulz, 2009; Schulz and Laessle, 2012). Effects of acute stress on overall food intake in BED are inconsistent, with some studies showing increased overall intake compared with non-BED after acute stress (Lyu and Jackson, 2016) but with most studies finding no differences (Laessle and Schulz, 2009; Schulz and Laessle, 2012; Klatzkin et al., 2018). A possible explanation for the inconsistent results in food intake might be the lab setting and the stress tasks used. Whilst it has not been investigated in BN/BED, overweight subjects eat less in social settings compared with being alone, this could be more pronounced in BN/BED because of negative emotions such as shame (Krantz, 1979; Salvy et al., 2007). The stress tasks also differed between studies. Physical and psychological stressors activate the various stress systems to different degrees (McRae et al., 2006). A physical stressor results in rapid activation of the autonomic nervous system via reflexive mechanisms in brainstem and hypothalamus, while a psychological stressor requires processing in the PFC and in turn activates the slower HPA axis via connections with limbic structures. Related to this, the timing of the food consumption after stress was also different between studies with some measuring before and other during the cortisol peak (i.e., 20–40 min after stress onset; Dickerson and Kemeny, 2004).

Many studies have also investigated heart-rate variability (HRV), blood pressure, salivary α-amylase, and cortisol responses to acute stress (Culbert et al., 2016; Peschel et al., 2016; Carroll et al., 2017; Wierenga et al., 2018; Naishe et al., 2019). HRV responses in BN/BED are not affected by acute stress and seem to not display the decrease during acute stress and increase poststress normally found in HC, although baseline levels are higher in BN/BED (Murialdo et al., 2007; Messerli-Bürgy et al., 2010; Hilbert et al., 2011; Het et al., 2015a). Blood pressure increase in response to acute stress was shown to be blunted in BN subjects compared with HC (Koo-Loeb et al., 1998; Ginty et al., 2012). Similarly, the majority of studies report a blunted cortisol response in BN and BED following acute stress (Koo-Loeb et al., 1998; Ginty et al., 2012; Rosenberg et al., 2013; Het et al., 2015b; Culbert et al., 2016; Carnell et al., 2018; Vaz-Leal et al., 2018; Wierenga et al., 2018; Naishe et al., 2019) but not all do (Coutinho et al., 2007; Vannucci et al., 2015). Interestingly, the blunted cortisol reactivity was positively associated with binge-eating severity in BED (Coutinho et al., 2007) and an increase in desire to eat after acute stress exposure (Rosenberg et al., 2013). Some have hypothesized that the blunted cortisol reactivity could be caused by long-term HPA axis activation (Het et al., 2015b; Culbert et al., 2016) because of high levels of chronic stress. Baseline HRV differences largely point to increased parasympathetic activity (Peschel et al., 2016), suggesting a blunted influence of the HPA axis. In addition, this idea is supported by the findings of increased baseline cortisol levels in BN (Monteleone et al., 2017; for an overview, see Culbert et al., 2016). However, baseline cortisol results are rather inconclusive, with only some suggesting a decrease in baseline cortisol levels and cortisol awakening response (Rosenberg et al., 2013; Vaz-Leal et al., 2018).

To summarize, most studies suggest an overall blunted physiological effect of stress in BN and BED, both after acute stress induction and in resting baseline measurements. This largely seems to be related to higher chronic stress levels and negative affect in these disorders. Although lab studies do not show a behavioral link between food intake and acute stress, they are related physiologically. Acute stress elicits an increase in salivary ghrelin, a peptide that promotes food intake in BN compared with non-BN (Monteleone et al., 2012; Monteleone and Maj, 2013; Gluck et al., 2014). Other studies confirm the importance of hormonal responses in eating behavior, including insulin, leptin, and extrahypothalamic corticotrophin releasing factor (CRF; for an overview, see Schepers and Markus, 2015; Sinha, 2018). Similar to the effects of acute stress, it is proposed that in healthy individuals CRF suppresses appetite during and directly after a stressful event. Subsequently, glucocorticoids stimulate feelings of hunger to offset the expanded energy. Chronic stress on the other hand appears to be able to expose the individual to long-term heightened levels of glucocorticoids, thereby increasing ghrelin levels and stimulating eating behavior of high caloric foods through reward sensitivity (Sominsky and Spencer, 2014; Schepers and Markus, 2015; Sinha, 2018). These effects on reward sensitivity by metabolic state can also potentially drive individuals to make more risky (variable outcome) choices when hungry (Symmonds et al., 2010).

#### Functional imaging studies

**Figure 3.**
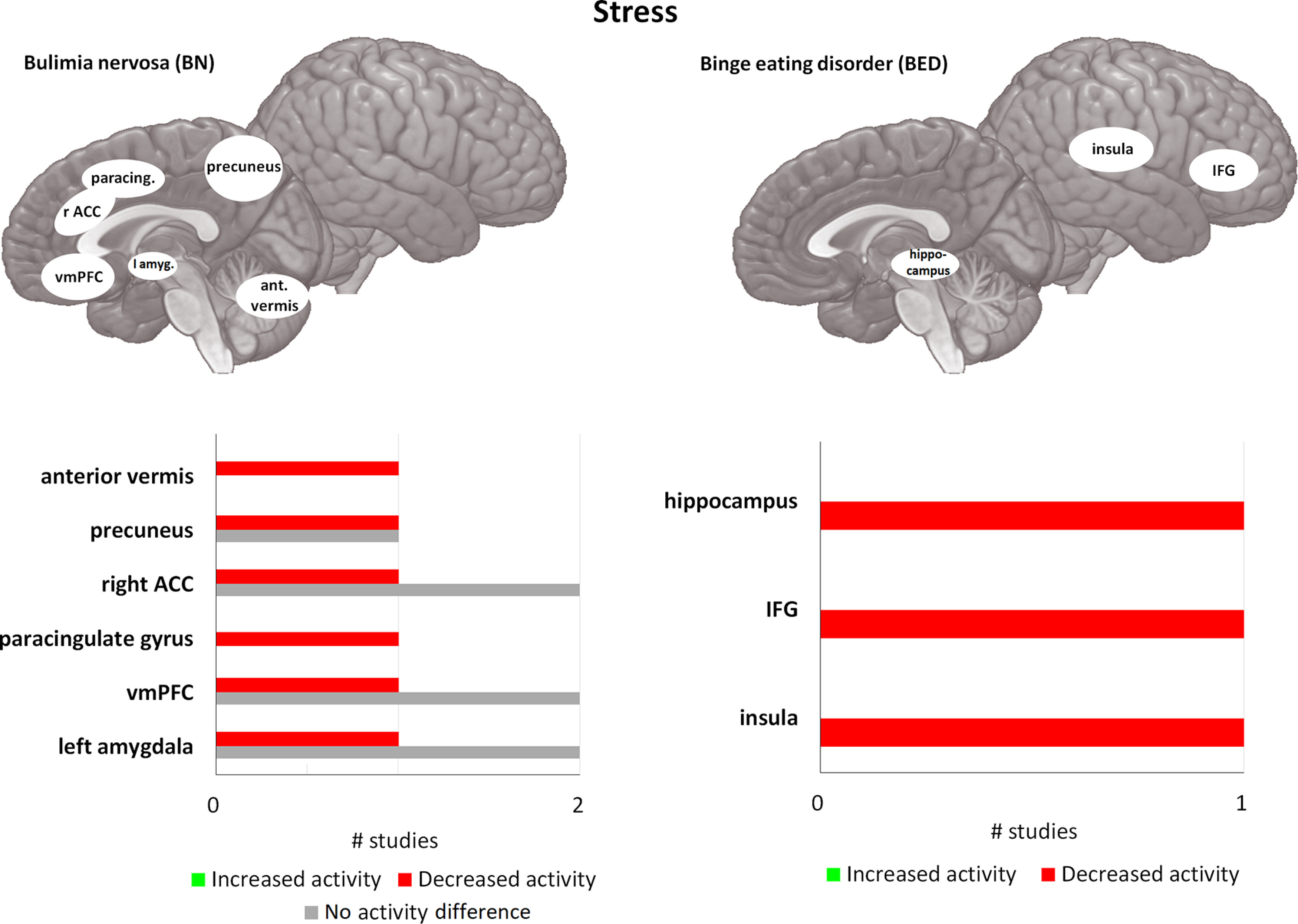
Differences in activity in BN and BED under acute stress/negative affect, using passive food viewing tasks, compared with matched controls. For each area, the bar graph indicates the number of studies that found a decrease in activity (= red), or an increase in activity (= green), and the studies that found no difference in activity (= gray), during passive food viewing tasks under acute stress. All of the included studies found at least one brain area that was significantly different in activation in BN and/or BED compared with HC. vmPFC = ventromedial prefrontal cortex, amyg. = amygdala, ant. vermis = anterior vermis of cerebellum, ACC = anterior cingulate cortex, paracing. = paracingulate gyrus, IFG = inferior frontal gyrus. If no indication of lateralization is given (either left or right), differences are observed bilaterally.

Acute stress and the processing of food stimuli appear to be associated, as reward sensitivity is shown to be altered in BN and BED after acute stress exposure (see Table 1; Fig. 3; Lyu and Jackson, 2016; Collins et al., 2017; Fischer et al., 2017; Wonderlich et al., 2018). Relevant brain areas seem to largely overlap with the network involved in reward sensitivity when not under acute stress. As mentioned previously, the cingulate cortex and insula are involved in processing reward values (Alexander and Brown, 2019; Rolls, 2019) and have been observed to be affected by acute stress (van Ruitenbeek et al., 2021). Other areas like the amygdala and hippocampus are commonly known to regulate stress. Specifically, the hippocampus is associated with inhibition of the HPA axis (Ulrich-Lai and Herman, 2009) and the amygdala is well-known to respond during stress (Marle et al., 2009; for an overview, see Zhang et al., 2018). In addition, these areas project to several relevant areas for reward sensitivity (Salzman and Fusi, 2010; Abivardi and Bach, 2017). In support of the overlap, they have been implicated in reward sensitivity tasks without acute stress manipulation (Filbey et al., 2012).

When comparing prestress with poststress reward processing in BN compared with HC, a decrease in activation is predominantly reported in the vmPFC, ACC, precuneus, and amygdala in BN patients (Collins et al., 2017; Fischer et al., 2017; Smith et al., 2018). Furthermore, the decrease in activity prestress to poststress in the bilateral precuneus, ACC, and dlPFC during a reward sensitivity task was associated with higher subjective stress before binge eating episodes in daily life (Fischer et al., 2017). On the other hand, amygdala and the vmPFC activation did not moderate subjective stress levels before binge eating episodes (Fischer et al., 2017). However, another study showed that activation in the bilateral vmPFC and right amygdala moderated negative affect prebinge and postbinge episode in BN (Wonderlich et al., 2018). Larger decreases in activity in these areas were associated with larger increases in negative affect before a binge eating episode. Conversely, positive affect prebinge and postbinge eating episode was moderated by activation in the right ACC and bilateral vmPFC, with larger decreases in activity also being associated with larger increases in positive affect after a binge eating episode. Combined, these results suggest that the vmPFC, ACC, amygdala, precuneus, and dlPFC play a role in regulating negative and positive affect before and after a binge eating episode. Indeed, the role of the vmPFC in successfully regulating negative affect has also been demonstrated in healthy samples; with larger decreases in activation following acute stress being reflective of a reduced coping and a higher frequency of maladaptive coping behaviors, including emotional eating (Sinha et al., 2016). Two independent samples of BN showed reduced activity in the precuneus, paracingulate gyrus, and anterior vermis of the cerebellum, while HC showed an increased activity from prestress to poststress on reward sensitivity (Collins et al., 2017). Furthermore, decreased activation in the hippocampus in BED compared with non-BED was found for showing high-caloric and low-caloric foods compared with neutral images, and the decrease was largest in response to high-caloric food images (Lyu and Jackson, 2016). Interestingly, reduced hippocampal activation predicted larger amounts of chocolate intake after the fMRI scans in the entire sample (Lyu and Jackson, 2016).

Taken together, these results show that several networks are affected prestress to poststress in BN/BED during reward processing. In BN, there is support for a decrease in activation poststress in the vmPFC, amygdala, precuneus, and ACC. Decrease in these areas are also associated with increased negative affect prestress, and positive affect poststress. So far, only one study has investigated reward sensitivity after acute stress exposure in BED (Lyu and Jackson, 2016), suggesting that the hippocampus may play an inhibitory role in reward sensitivity after acute stress. Overall, then, the results from these studies suggest that the vmPFC and ACC are likely to be involved in regulating negative affect, in addition to their role in reward sensitivity and cognitive control. Crucially, the ventral striatum and OFC, which are involved in processing of reward values, do not seem to be affected in reward processing poststress in BED/BD. A blunted stress response is confirmed by a number of different measures, including salivary cortisol, blood pressure and brain activity. The blunted stress response might disproportionally be associated with an increased drive for binging and is likely reflective of prolonged activation of the HPA axis and autonomic nervous system. Several studies (Carrard et al., 2012; Haedt-Matt and Keel, 2011; Fischer et al., 2018) suggest that the link between acute stress, negative affect, and eating behavior outside the lab is strong, although lab studies could not find differences in food intake.

## Brain Differences in BN/BED

### Volumetric differences in GM

**Figure 4.**
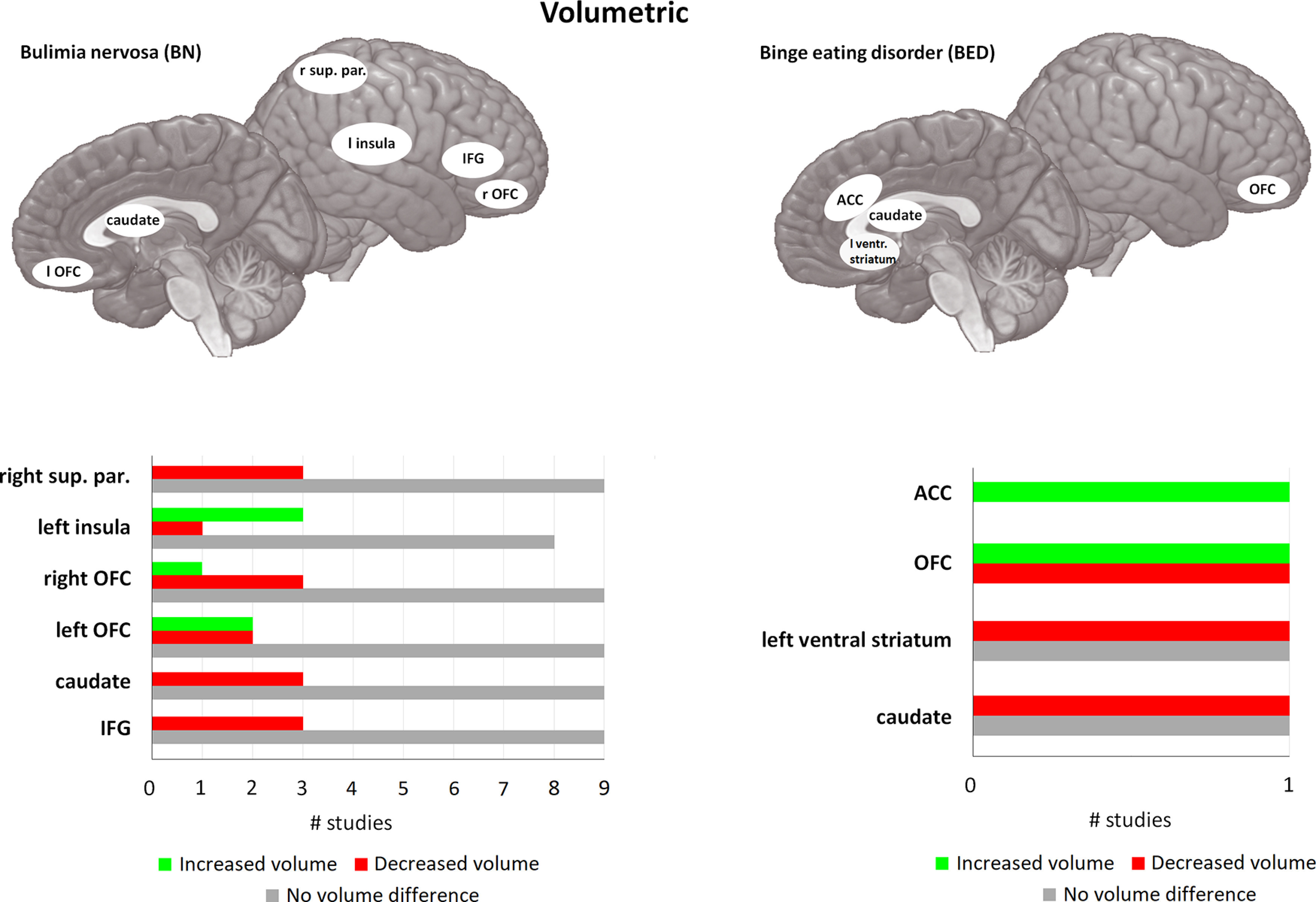
Volumetric differences in BN and BED compared with matched controls. For each area, the bar graph indicates the number of studies that found a reduction in volume (= red), or an increase in volume (= green), and the studies that found no difference in activity (= gray). For BN, 10 of the 14 included studies found at least one brain area that was significantly different compared with HC. Four studies did not find any significant differences between BN and HC. caudate = caudate nucleus, OFC = orbitofrontal cortex, sup. par. = superior parietal cortex, IFG = inferior frontal gyrus, ventr. striatum = ventral striatum, ACC = anterior cingulate cortex. If no indication of lateralization is given (either left or right), differences are observed bilateral. For the left part (BN) of this figure only, areas with one study indicating differences are not displayed, because of the large number of areas found in BN. For the right part (BED) all studies are displayed. For a full overview for differences in BN, please see Table 2.

Neuroscientific studies investigating volumetric GM differences between BN subjects and HC reveal specific volume reductions and increases in cortical and subcortical structures (for an overview, see Fig. 4 and Table 2), which reflect those part of the networks associated with the three cognitive constructs discussed above in Cognitive Constructs Associated with Binging Behavior. In both BN and BED, several structures associated with reward processing were shown to be affected, such as the ventral and dorsal striatum (Schäfer et al., 2010; Amianto et al., 2013a; Frank et al., 2013; Coutinho et al., 2015; Voon et al., 2015). Both the ventral and dorsal striatum comprise multiple substructures that are associated with processing different kinds of information. Therefore, identifying which structures are affected is important to determine potential functional roles. The ventral striatum is comprised of the olfactory tubercle and nucleus accumbens, the dorsal striatum is comprised of the putamen and caudate nucleus (Meredith et al., 2008). For the dorsal striatum, GM volume in the bilateral caudate was shown to be reduced in BN compared with HC, with a larger reduction in the right hemisphere (Amianto et al., 2013a; Coutinho et al., 2015) and particularly in the dorsal caudate nucleus (Frank et al., 2013). Differences in GM of the putamen seem even more lateralized, with BN showing increased volume in the left putamen compared with HC (Amianto et al., 2013a) but reduced volume in the right dorsal putamen (Frank et al., 2013). Slightly different patterns are observed in BED (Schäfer et al., 2010; Voon et al., 2015), but the number of studies is limited. A reduction in GM volume was only observed in the bilateral caudate nucleus, no differences between BED and HC were found in putamen volume (Schäfer et al., 2010; Frank et al., 2013; Coutinho et al., 2015; Voon et al., 2015). A study directly comparing BED, BN, and HC subjects showed that BN subjects had greater GM volume in the dorsal striatum than BED subjects, suggesting that it might be more affected in BN (Schäfer et al., 2010). For the ventral striatum, specifically for the nucleus accumbens, differences in volume seem to be relatively inconsistent. Some studies indicate an increase in accumbal volume in BN compared with HC and BED (Schäfer et al., 2010; but see Coutinho et al., 2015), while one study indicated that GM volume was reduced in BED in the bilateral nucleus accumbens (Voon et al., 2015).

**Table 2:** Overview of publications on volumetric, functional connectivity, and structural connectivity in BN and BED

Study	Subjects	Gender (♀/♂)	Age $\bar{\text{x}}$ (SD)	BMI $\bar{\text{x}}$ (SD)	Diagnosis	Method	Findings
Volumetric							
BN							
Amianto et al. (2013a)*^g^*	BN (13)	13/0	22 (3)	22 (2)	SCID DSM-IV-TR	VBM	BN>HC, increased volume GM: left PCL, precuneus, left putamen, left insula
	HC (14)	14/0	24 (3)	21 (2)			HC>BN, reduced volume GM: caudate, right thalamus
Berner et al. (2018)***^g^***	BN and BN-spectrum (60)	60/0	18.8 (4.1)	22.4 (2.4)	Patients, N.R.	Cortical thickness, ROI	BN>HC, increased Cortical thickness: left ventral PCC
	HC (54)	54/0	19.2 (5.5)	21.9 (2.1)			HC>BN, reduced Cortical thickness: pars triangularis, right SPC, left dorsal PCC
Berner et al. (2019)***^g^***	BN and BN-spectrum (62)	62/0	18.8 (4)	22.4 (2.5)	SCID DSM-IV-TR, EDE	VBM	HC>BN, inward deformations (vertex indices) right anterior lateral and medial pallidum; internal and external GP
	HC (65)	65/0	19.3 (5.7)	22.6 (2.8)			N.S.
Canna et al. (2017)***^g^***	BN (13)	13/0	27.2 (2)	N.R.	Patients, SCID DSM-5	VBM	N.S.
	HC (16)	16/0	26.1 (3.5)	21.1 (1.6)			
Coutinho et al. (2015)***^g^***	BN (21)	21/0	31.57 (8.27)	21.39 (2.36)	Patients, DSM-IV	Drawn ROIs	HC>BN, reduced volume GM: caudate nucleus
	HC (20)	20/0	30.9 (8.79)	22.11 (3.2)			
Cyr et al. (2017)*^f^	BN (33)	33/0	16.5 (1.5)–18.1 (1.5)–19.3 (1.5)	22.1 (2.8)–23.2 (2.8)–23.8 (2.7)	Patients, 22 DSM-5, 11 OSFED-BN	Cortical thickness, ROI, follow-up	HC>BN, reduced thickness Right inferior frontal gyrus pars opercularis and pars orbitalis (OFC), consistent over all time points IFG opercularis on baseline, 12 months, 24 months, but not 36 months follow-up (N.S.)
	HC (28)	28/0	16.2 (2.1) –17.3 (2.1)–18.8 (2.3)	21.4 (3.5)–22.6 (3.5)–23.9 (4.9)			Correlations Thickness frontal pole correlated negatively with objective binge eating episode frequency, baseline only Average thickness lateral OFC and IFC orbitalis correlated negatively with frequency vomiting, over all time points
Frank et al. (2013)***^g^***	BN (19)	19/0	25.2 (5.3)	22.6 (5.7)	Patients, SCID DSM-IV	VBM	BN>HC, increased volume GM: left OFC, left anterior ventral insula, GR
	HC (24)	24/0	27.4 (6.3)	21.6 (1.3)			HC>BN, reduced volume GM: dorsal caudate, right dorsal putamen WM: right MTL, right IFG
Joos et al. (2010)***^g^***	BN (17)	17/0	24.5 (4.8)	21.1 (2.5)	DSM-IV	VBM	N.S.
	HC (18)	18/0	26.9 (5.7)	21.2 (2)			
Marsh et al. (2015)***^g^***	BN (34)	34/0	21.6 (6)	22.1 (2)	SCID DSM-IV-TR	Cortical thickness	BN>HC, increased volume GM: MOG, IOG, LG, right IPL WM: reflective of these results
	HC (34)	34/0	22.08 (6.5)	22.13 (2.3)			HC>BN, reduced volume GM: MFG, PreCG, ITG, PCC, right PostCG, right SPG, right cuneus, left IFG, left LSG, left precuneus, left FG WM: reflective of these results
Mettler et al. (2013)***^g^***	BN (20)	20/0	25.2 (5.3)	22.59 (5.69)	Patients, DSM-IV-TR	VBM	N.S.
	HC (21)	21/0	27.5 (6.6)	21.55 (1.19)			
Wagner et al. (2006)***^f^***	BN, recovered (10)	10/0	24 (6.1)	23.1 (2.4)	Ex-patients, recovery 29.8 (18.1) months	VBM	N.S. When covarying for age, BN>HC GM insula
	HC (31)	31/0	26.8 (7.3)	21.9 (2)			
Wallace et al. (2020)***^f^***	BN-spectrum (247)	169/78	19.43 (1.23)	N.R.	EDI-3 Bulimia subtest, score >0	Cortical thickness	Negative correlation with bulimia scores in thickness GM: OFC, insula, left S1/S2, left IPC
Westwater et al. (2018)***^g^***	BN and BN-spectrum (33)	33/0	22.6 (4.13)	23.9 (3.1)	N.R., EDE, EDE-Q	Cortical thickness, and per vertex connectivity	Negative correlation EDE-Q BN symptoms with cortical thickness GM: rPostCG, right rostral MFG, rITG, rSFG, lOFC, lIPC, lMTG, left caudal MFG Areas with reduced cortical thickness show increase in structural connectivity estimation (per vertex, T1 MPRAGE)
BN+BED							
Voon et al. (2015)***^g^***	BED (20)	12/8	43.95 (9.47)	34.12 (5.49)	DMS-IV-TR	VBM, ROI	HC>BED, reduced volume GM: medial OFC, caudate, left VS, left lateral OFC
	OB (20)	9/11	44.7 (10.12)	32.88 (3.53)			
Schafer et al. (2010)***^g^***	BED (17)	17/0	26.4 (6.4)	32.2 (4)	DSM-IV-TR	VBM	BED>HC, increased volume GM: ACC, medial OFC
	BN (14)	14/0	23.1 (3.8)	22.1 (2.5)			BN>HC, increased volume GM: medial OFC, VS
	HC (19)	19/0	22.3 (2.6)	21.7 (1.4)			BN>BED, volume GM: VS, DS, left lateral OFC, left medial OFC
Structural connectivity**							
BN							
Canna et al. (2017)***^g^***	BN (13)	13/0	27.2 (2)	N.R.	Patients, SCID DSM-5	DTI	FA: N.S.
	HC (16)	16/0	26.1 (3.5)	21.1 (1.6)			
Frank et al. (2016)***^g^***	BN (25)	25/0	24.64 (4.22)	23.56 (5.89)	Patients, SCID DSM-IV	Diffusion, PFT	BN>HC, increased connectivity, left hemisphere pI an dAI to: medial PFC, middle OFC, VS vAI to: middle OFC, VS VS to: inferior OFC, GR
	HC (26)	26/0	24.39 (3.49)	21.61 (1.21)			BN>HC, increased connectivity, right hemisphere pI to: VS dAI to: medial PFC, middle OFC VS to: medial OFC
							HC>BN, reduced, left hemisphere vAI to: inferior OFC, CeA Hypothalamus to: middle OFC Medial PFC to: GR
							HC>BN, reduced, right hemisphere BLA to: dAI, VS CeA to: hypothalamus
He et al. (2016)***^g^***	BN (28)	28/0	21.32 (6.11)	21.95 (2.13)	SCID DSM-IV-TR	DTI, TBSS	HC>BN, reduced FA: FMajor, Fminor, SLF, IFOF, ATR, CST, UF, cingulate gyrus
	HC (28)	28/0	20.61 (6.12)	22.18 (2.14)			BN>HC, increased RD: FMajor, Fminor, IFOF, ATR, CST, cingulate gyrus, left SLF
Mettler et al. (2013)***^g^***	BN (20)	20/0	25.2 (5.3)	22.59 (5.69)	Patients, DSM-IV-TR	DTI	BN>HC, increased ADC: CR into ALIC, corpus callosum, left IFOF, left UF, left EC
	HC (21)	21/0	27.5 (6.6)	21.55 (1.19)			HC>BN, reduced FA: CR into PLIC, corpus callosum
Wang et al. (2019)***^g^***	BN (48)	48/0	22.0 (3.4)	21.0 (2.6)	Patients, DSM-IV, MINI	DTI, nodal, NBS	BN>HC, increased Nodal strength: left superior OFC, ITG, insula, hippocampus, PHG, thalamus Local efficiency: left superior OFC, STG, ITG, superior temporal pole, thalamus, amygdala
	HC (44)	44/0	23.1 (1.7)	20.5 (1.4)			HC>BN, reduced Nodal strength: left ACC, right precuneus Global efficiency: left GR, right OFC, insula, putamen, pallidum, amygdala, PreCG, PostCG, SMG, precuneus, FG Local efficiency: right PreCG, precuneus
BED							
Estella et al. (2020)***^g^***	BED (17)	17/0	33.82 (7.2)	36.07 (0.8)	DSM-5, EDE	DTI, TBSS	BED>OB, increased FA: Fminor AD: left SLF, cingulum (ACC, PCC), corpus callosum
	OB (BED controls; 13)	13/0	38.03 (9.7)	33.64 (4.7)			BED>HC, increased AD: Right Fminor, SLF Left ILF, Fmajor, IFOF Bilateral ATR
	HC (non-OB; 17)	17/0	34.70 (11.0)	22.50 (2.0)			
Functional connectivity							
BN							
Amianto et al. (2013b)*^g^*	BN (12)	12/0	23 (5)	21.57 (2.38)	Patients, DSM-IV	RS, ICA	BN>HC, increased connectivity Cerebellum (IX, X) to: left insula, TL Lateral cerebellum, ACC, precuneus
	HC (10)	10/0	24 (3)	21.35 (3.16)			HC>BN, reduced connectivity Cerebellum (IX, X) with PL Right IFG
Canna et al. (2017)***^g^***	BN (13)	13/0	27.2 (2)	N.R.	Patients, SCID DSM-5	RS, VMHC	HC>BN, reduced VMHC: FL, from OFC to dlPFC Coherence: Slow-4 and Slow-5 for OFC-dlPFC cluster
	HC (16)	16/0	26.1 (3.5)	21.1 (1.6)			
Lee et al. (2014)***^g^***	BN (20)	20/0	22.9 (3.9)	21.6 (2.3)	Patients, SCID, DSM-IV	RS, ROI, seed-based	BN>HC, increased synchrony dACC to: left OFC, right precuneus, medial OFC
	HC (20)	20/0	23.3 (1.8)	19.9 (1.9)			HC>BN, reduced synchrony dACC to: left PHG
Spalatro et al. (2019)***^f^***	BN (16)	16/0	21.56 (2.35)	21.84 (2.35)	Patients, SCID DSM-IV-TR and DSM-5	RS, ROI	BN>HC, increased neural variability (*SD*) Slow-4: ventral attention network Slow-5: N.S.
	HC (17)	17/0	23.27 (2.19)	21.42 (1.85)			
Wang et al. (2020)***^g^***	BN (51)	51/0	N.S.	20.8 (2.2)	Patients, DSM-IV, MINI	RS, ROI, seed-based	BN>HC, increased connectivity Right DC to bilateral putamen, GP, caudate, thalamus Putamen (DCP, DRP, VRP) to thalamus, GP, putamen
	HC (53)	53/0	N.S.	20.4 (1.7)			HC>BN, reduced connectivity VS to PreCG, PostCG, OG Putamen (DCP, DRP, VRP) to right SFG/MFG
BED							
Oliva et al. (2019)*^f^*	BED and BED-spectrum (19)	15/4	23.89 (3.4)	22.53 (2.04)	Eating attitude test (EAT-26), >1 episode per month	RS, ROI, seed-based	BED>HC, increased N.S. for seeds left putamen, ITG, SPL N.S. correlation with impulsivity scores
	HC (20)	15/5	25.31 (3.2)	21.25 (2.07)			HC>BED, reduced Degree centrality: right MFG, left MTL/ITL, SPL, insula Connectivity: right MFG to right anterior insula, rMFG to rMFG/IFG
BN+BED							
Stopyra et al. (2019)***^g^***	BN (29)	29/0	27.45 (10.55)	21.33 (2.99)	Patients, SCID DSM-IV	RS, ICA, ROI, seed-based	BN>HC, increased connectivity ICA: right dorsal medial PFC
	HC (BN controls; 30)	30/0	26.86 (6.59)	21.85 (1.80)			OB>BED, reduced connectivity ICA: right medial dACC, right dorsal medial PFC
	BED (27)	23/4	38.39 (13.06)	32.64 (4.13)			BED>OB, increased connectivity seed-based with dACC: right cerebellum, right LG
	OB (BED controls; 28)	24/4	39.40 (10.48)	33.58 (4.54)			HC>BN, reduced connectivity ICA: left medial dACC, left ventral medial PFC
							BN>BED ICA: right dorsal medial PFC, left MFG, left AG seed-based with dACC: RSC
							BED>BN ICA: left medial dorsal PCC seed-based with dACC: left PreCG, right PostCG, left SMA

Abbreviations general: N.S. = not significant, N.R. = not reported.

Abbreviations subjects: BN = bulimia nervosa, BED = binge eating disorder, HC = healthy control, OB = obese control, EDIBul = bulimia symptoms on eating disorder inventory.

Abbreviations diagnosis: SCID = structured clinical interview for the DSM, DSM = diagnostic and statistical manual of mental disorders, DSM-IV-TR = DSM IV textual revision, OSFED = other specified feeding or eating disorders, EDE = eating disorder examination interview, EDI = eating disorder inventory.

Abbreviations method: VBM = voxel-based morphometry, ROI = region-of-interest analysis, Ica = independent component analysis, RS = resting state, VMHC = voxel-mirrored homotopic connectivity, DTI = diffusion tensor imaging, TBSS = tract-based spatial statistics, PFT = probabilistic fiber tractography, NBS = network-based statistic.

Abbreviations findings general: GM = gray matter, WM = white matter, FA = fractional anisotropy, ADC = apparent diffusion coefficient, RD = radial diffusivity.

Abbreviations findings gyri: MOG = middle occipital gyrus, IOG = inferior occipital gyrus, LG = lingual gyrus, AG = angular gyrus, MFG = middle frontal gyrus, IFG = inferior frontal gyrus, SFG = superior frontal gyrus, PreCG = precentral gyrus, PostCG = postcentral gyrus, ITG = inferior temporal gyrus, MTG = middle temporal gyrus, STG = superior temporal gyrus, SPG superior parietal gyrus, LSG = lateral superior gyrus, FG = fusiform gyrus, PHG = parahippocampal gyrus, GR = gyrus rectus, SMG = supramarginal gyrus.

Abbreviations findings cortex: PCC = posterior cingulate cortex, (d)ACC = (dorsal) anterior cingulate cortex, OFC = orbitofrontal cortex, PFC = prefrontal cortex, SPC = superior parietal cortex, RSC = retrosplenial cortex, S1/S2 = somatosensory cortex, IPC = inferior parietal cortex.

Abbreviations findings other areas: SMA = supplementary motor area, PCL = paracentral lobule, TL = temporal lobe, PL = parietal lobe, FL = frontal lobe, MTL = medial temporal lobe, ITL = inferior temporal lobe, IPL = inferior parietal lobe, SPL = superior parietal lobe, VS = ventral striatum, DS = dorsal striatum, DC = dorsal caudate, GP = globus pallidus, DCP = dorsal caudal putamen, DRP = dorsal rostral put., VRP = ventral rostral put., CeA = central nucleus amygdala, BLA = basolateral amygdala, pI = posterior insula, dAI = dorsal anterior insula, vAI = ventral anterior insula, Cerebellum (IX,X) = vermis and paravermis of cerebellum, lobule IX/X.

Abbreviations findings WM: SLF = superior longitudinal fasciculus, ILF = inferior longitudinal fasciculus, IFOF = inferior fronto-occipital fasciculus, UF = uncinate fasciculus, ATR = anterior thalamic radiation, CST = corticospinal tract, FMajor = major forceps, Fminor = minor forceps, CR = corona radiata, ALIC = anterior limb of internal capsule, PLIC = posterior limb of internal capsule, EC = external capsule. *Age and BMI are displayed as baseline, follow-up 1, and follow-up 2. ** Structural connectivity can be measured with a number of different techniques (e.g., diffusion tensor imaging, probability tracking, TBSS), and do not necessarily give perfectly comparable results. Values such as FA and MD are not specific (increase in FA and decrease in MD reflects reduction in WM integrity), and differences could be attributed to myelin integrity, axonal diameter, axonal density, less coherent orientation of axons, etc. Higher RD is however more specific to myelin loss, and lower AD to axonal degradation (although there are exceptions; see Aung et al., 2013; Solowij et al., 2017). *^g^* and *^f^* refer to the quality assessment done by two independent raters (for more details, see Concluding Remarks, Quality assessment). Represents a good (≥7.5) or a fair (4–7.5; out of 10) rating, respectively.

Differences in volume of cortical areas have also been reported (Wagner et al., 2006; Frank et al., 2013; Berner et al., 2019; Wallace et al., 2020), although findings are not always consistent with each other (Amianto et al., 2013a; Marsh et al., 2015). One of these areas is the insula, which is associated with a large number of functions (e.g., perception, self-reflection, cognitive functioning, emotion, homeostasis) and is shown to be involved in a wide variety of tasks (Uddin et al., 2017). Insula GM volume seems particularly affected in BN, with studies predominantly showing an increase in GM volume compared with HC (Amianto et al., 2013a; Frank et al., 2013). These differences seem to persist to some extent after recovery, with recovered BN subjects showing an increase in volume when age was controlled for (Wagner et al., 2006). However, larger insula volume might be limited to patients only, as a large subclinical group with limited BN symptoms showed a negative association between BN subscale scores (Eating Disorder Inventory, EDI-3) and GM thickness of the bilateral insula and the left inferior parietal cortex (Wallace et al., 2020). Indeed, parts of the parietal cortex are also affected in BN with most consistent findings in the inferior parietal cortex. Reduction in volume is observed of the right inferior parietal lobule (Marsh et al., 2015), but also in the right superior parietal cortex (Marsh et al., 2015; Berner et al., 2018), and both an increase and reduction in volume in the precuneus (Amianto et al., 2013a; Marsh et al., 2015). In addition, BN symptom severity (Eating Disorder Examination Questionnaire, EDE-Q) was shown to be negatively associated with cortical thickness in parts of the inferior parietal cortex and temporal cortex (Westwater et al., 2018). The ACC seems uniquely increased in GM volume in BED compared with HC (Schäfer et al., 2010), although the PCC was found to be reduced in GM volume in BN (Marsh et al., 2015; Berner et al., 2019).

Frontal areas, and in particular the OFC, are associated with reward processing and show GM differences between BN and HC, and BED and HC. Several studies show an increase in GM volume in BN compared with HC, in particular in the bilateral medial OFC (Schäfer et al., 2010; Frank et al., 2013; Cyr et al., 2017). In contrast, OFC GM thickness was shown to be negatively associated with bulimia subscale scores (EDI-3) and BN symptoms (EDE-Q) in subclinical populations (Westwater et al., 2018; Wallace et al., 2020), and the number of vomiting episodes in BN subjects was negatively associated with lateral OFC GM volume (Cyr et al., 2017). Some studies showed that several other parts of the PFC were also affected, such as the IFG (Marsh et al., 2015; Cyr et al., 2017). Likewise, BN symptoms (EDE-Q) were found to be negatively associated with cortical thickness in caudal and rostral parts of the middle frontal gyrus (MFG) and the superior frontal gyrus (Westwater et al., 2018). In addition, BED patients also showed reduced left lateral OFC volume compared with HC (Schäfer et al., 2010; Voon et al., 2015). Results concerning medial OFC volume are however inconsistent, with one study finding an increase in volume, and another finding a reduction in volume (Schäfer et al., 2010; Voon et al., 2015).

To summarize, GM volumetric differences between BED and HC and especially between BN and HC are consistently found in the OFC, striatum, and insula (see Fig. 4). These are associated with a number of functions that are associated with BN and BED pathology (Yan et al., 2016; Uddin et al., 2017; Stalnaker et al., 2018; Setogawa et al., 2019). Specifically, these areas show considerable overlap with those presented in the previous section; areas associated with reward sensitivity, cognitive control, and negative affect (see above, Cognitive Constructs Associated with Binging Behavior). We will integrate these findings below, see Concluding Remarks. Interestingly, some of the discussed studies (Frank et al., 2013; Marsh et al., 2015) indicate that GM volumetric differences in a number of cortical areas are related to the underlying WM connectivity, particularly in the parietal cortex. In further support, Westwater et al. (2018) observed that while BN symptoms were negatively associated with cortical thickness in a number of areas (e.g., OFC, frontal gyrus, superior parietal cortex), the areas also showed an increase in estimation of WM connectivity (Westwater et al., 2018). In general, brain areas and their connections are organized into networks, and this connectivity plays a crucial role in information processing in the brain. Volumetric differences in the WM that connects these areas likely affect the functioning of these connections. Subsequently, connectivity studies will be informative to determine whether these are also affected in BN and BED, and what role they might play in these pathologies.

### Structural connectivity

Brain regions are physically connected via WM tracts, typically measured using diffusion weighted imaging (Fields, 2008). Differences in this structural connectivity between BN and HC reflect the GM volumetric differences, as the connectivity between these areas is particularly affected (see Table 2; i.e., connections between parts of the ventral and dorsal striatum, insula, (pre)frontal cortex, amygdala). For example, compared with HC, BN patients showed reduced connectivity in the bilateral corona radiata extending in the posterior and anterior limb of the internal capsule (Mettler et al., 2013; He et al., 2016), which lies between the caudate nucleus and putamen. Connections of the ventral striatum with both cortical and subcortical areas are also affected. For example, connectivity between the ventral striatum and the insula and the frontal cortex (inferior and medial OFC) was shown to be increased in BN compared with HC (Frank et al., 2016; Wang et al., 2019), while connectivity between the ventral striatum and the right basolateral amygdala (BLA) was reduced (Frank et al., 2016). Network analysis in BN compared with HC shows that mesocorticolimbic pathways and other subcortical connections in the right hemisphere might be negatively affected in global efficiency (Wang et al., 2019), a measure associated with parallel information processing (Bullmore and Sporns, 2012). Pathways in the left hemisphere seem largely unaffected or even increased (Wang et al., 2019). This fits with the volumetric differences discussed above, see Volumetric differences in GM, which show reductions in volume in the right hemisphere but some increases in the left hemisphere.

Connectivity of the insula also seems affected in BN. Overall the left insula shows increased connectivity (Wang et al., 2019), corresponding with the increase in volume. However, depending on the projection, some WM tracts are increased in connectivity, and others decreased. Particularly, WM tracts between the anterior insula and cortical areas show increased connectivity, while decreased connectivity is observed between the anterior insula and subcortical areas (Frank et al., 2016). Specifically, connectivity was increased between the bilateral anterior insula and the medial PFC and the middle OFC, but was reduced between the right anterior insula and the BLA, and the left anterior insula to the central nucleus of the amygdala (CeA) and the inferior OFC (Frank et al., 2016). Some other areas show differences in connectivity as well, such as reduced connectivity from the left ACC, right precuneus, hypothalamus, but increased connectivity from the temporal cortex, hippocampus, and thalamus (Frank et al., 2016; Wang et al., 2019). One exception is the study by Canna et al. (2017) that found no differences in structural connectivity between BN and HC.

In long-range WM association fiber tracts that connect the frontal lobe with other lobes and subcortical areas, a reduction in connectivity was found in BN compared with HC (Mettler et al., 2013; He et al., 2016). Namely, connectivity differences were observed in the superior longitudinal fasciculus (SLF; Mettler et al., 2013; He et al., 2016), which predominantly connects the frontal cortex to the parietal cortex, while passing through the insula and around the putamen. Similarly, reduced connectivity was observed in BN in the bilateral inferior fronto-occipital fasciculus that connects the (pre)frontal cortex to the occipital cortex, and the uncinate fasciculus that connects the PFC to the amygdala, parahippocampus, and anterior temporal cortex (Mettler et al., 2013; He et al., 2016). This was also the case for numerous long range connections between the two hemispheres, with connectivity being reduced in BN compared with HC in the major and minor forceps, bilateral corpus callosum, and cingulate gyrus (Mettler et al., 2013; He et al., 2016). In contrast, a recent paper (Estella et al., 2020) showed that connectivity in some of these WM tracts appears to be increased in BED compared with HC and obese controls. Compared with obese controls, BED subjects showed increased connectivity in the minor forceps, corpus callosum, and cingulate gyrus (connecting the two hemispheres) and the SLF (connecting the frontal to parietal cortex). Some of the same results (forceps, SLF) were found when comparing BED and normal-weight HC (see Table 2), and when comparing obese controls to normal-weigh HC (Estella et al., 2020). This suggests that some of these differences might be related to obesity, rather than BED per se

To summarize, the observed WM tract differences echo those in GM volume and correspond to the networks associated with the three cognitive constructs. Differences in volume of the OFC, insula, and striatum fit well with the consistent differences in connection strength and/or microstructure between these areas. Indeed, the nature of some of the parameters that are affected (see Table 2) suggests that the differences might be relatively specific to myelin loss (although a loss of axons and/or a reduced density of axons is also possible; Aung et al., 2013; Solowij et al., 2017). Myelin forms a protective sheath around the axons of neurons, and by speeding up axon potentials, ensures normal cognitive and sensory function, being involved in several other psychiatric disorders as well (Fields, 2008). Compared with BN, BED subjects show an increase in connectivity rather than a decrease (Estella et al., 2020). More research is needed to confirm these findings and to elucidate the functional consequences for BN and BED.

### Functional connectivity

Functional connectivity is a measure of the functional association between brain areas operationalized by the temporal correlation or covariation of activity between those brain areas. Instead of structural images, functional connectivity uses fMRI, i.e., blood oxygenation level-dependent (BOLD) imaging to do so. Differences between BN/BED and HC in functional connectivity measures (see Table 2) largely overlap with those of structural connectivity, although not all do. Connectivity from the right dorsal caudate nucleus and putamen to other subcortical structures (putamen, globus pallidus, thalamus) was increased in BN compared with HC, compared with connectivity from the ventral striatum and putamen to cortical areas (central gyrus, occipital gyrus, superior frontal gyrus), which was shown to be reduced (Wang et al., 2020).

Connectivity of the parietal cortex seems to be largely reduced. For example, BN is shown to be characterized by a reduction in volume of GM and structural connectivity of the parietal cortex. Similarly, functional connectivity was reduced compared with HC within the inferior parietal cortex (Stopyra et al., 2019), within the temporo-parietal junction (TPJ; Spalatro et al., 2019), and between the parietal lobes and the cerebellum (Amianto et al., 2013b). In contrast, functional connectivity was shown to be increased in BN between the precuneus and cerebellum (Amianto et al., 2013b). BED subjects showed a similar pattern, with reduced connectivity strength compared with HC within the inferior parietal cortex (Stopyra et al., 2019) and less connectivity to the right superior parietal cortex (Oliva et al., 2020). Reduced connectivity compared with BN in the angular gyrus (Stopyra et al., 2019), and a reduction in connectivity to the right insula was also reported (Oliva et al., 2020).

In general, functional connectivity within and between frontal regions was found to be reduced in BN and BED compared with HC (Canna et al., 2017; Spalatro et al., 2019; Stopyra et al., 2019). Namely, reduced connectivity within the IFG (Spalatro et al., 2019) was found in BN compared with HC. Similarly, BN showed reduced connectivity within and between a cluster extending from the OFC to the dlPFC (Canna et al., 2017). Connectivity between the two hemispheres for this cluster was reduced as well (e.g., between the left IFG and right IFG; Canna et al., 2017). Interestingly, BN showed an increase in functional connectivity within the dorsomedial PFC (dmPFC), while BED showed a reduction in the dmPFC connectivity compared with HC (Stopyra et al., 2019). Moreover, when comparing BN and BED directly, BED also showed a reduction in functional connectivity compared with BN within the MFG (Stopyra et al., 2019), showing that areas in the frontal cortex are differently affected in BN and BED. Compared with HC, BED similarly showed less connectivity to the MFG and reduced connectivity strength from the right MFG to the right anterior insula and right IFG (Oliva et al., 2020). In the cingulate cortex, BED showed increased functional connectivity within the dorsal part of the PCC compared with BN (Stopyra et al., 2019). However, no differences for the PCC were found when comparing BED or BN with HC. For the ACC, BN and BED did show similar reductions in functional connectivity compared with HC (Stopyra et al., 2019). Connections between the ACC and left OFC, right precuneus, and cerebellum were only increased in BN (Amianto et al., 2013b; Lee et al., 2014). Connectivity between the ACC and parahippocampal gyrus was reduced compared with HC.

To summarize, numerous functional connectivity differences can be observed in BN and BED compared with HC. Most of these areas are also affected in terms of GM volume and structural connectivity, such as parietal areas, frontal areas (particularly the OFC), insula, and cingulate cortex. Only in BN an increase in functional connectivity between the insula and cerebellum was observed (Amianto et al., 2013b). It has to be mentioned that structural and functional connectivity are not always connected in a straightforward manner, although they are often positively correlated (see for example Uddin, 2013). This was confirmed by an animal study where severing connections between areas did not always lead to a change in functional connectivity (O’Reilly et al., 2013). Nevertheless, the overall patterns reflect similar differences between BED/BN and HC in GM volume, structural and functional connectivity.

## Concluding Remarks

In this review, we gave an overview of the brain networks associated with the three cognitive constructs most integral to BN and BED. Subsequently, we showed how these networks show an atypical brain organization, both in volume and connectivity compared with HC. We will now shortly summarize the findings presented in these sections, and explain how these differences in volume and connectivity may explain BED and BN symptomatology, and how this is likely involved in eliciting and maintaining binge eating.

### Reward sensitivity

Activation differences between BN/BED patients and HC evoked by tasks assessing reward sensitivity are not uniform. A distinction has to be made between passive viewing (anticipatory) tasks and actively receiving or consuming food (consummatory tasks). Both patient groups show a difference in activity in passive viewing tasks in the cingulate cortex (ACC and PCC), the ventral striatum, ventral tegmental area (VTA), and the insula (Schienle et al., 2009; Filbey et al., 2012; Balodis et al., 2013a, 2014; Oberndorfer et al., 2013; Simon et al., 2016). Generally, activity in the PCC is lower compared with HC, while activation is shown to be higher in the other areas (see Fig. 1). Activity differences in the medial OFC, that connects to the ventral striatum (nucleus accumbens), seem largely limited to BED in passive viewing tasks (Schienle et al., 2009). As mentioned previously, the ventral striatum is thought to play a role in reward prediction error, by comparing expected with received outcome values (Wang et al., 2016). The role of the medial OFC has been more controversial, especially when comparing it to the lateral OFC (Noonan et al., 2012). The medial OFC has been suggested to be involved value estimation (Sescousse et al., 2010; Stalnaker et al., 2018; Setogawa et al., 2019). However, it has been suggested that it is involved in a broader evaluation of value-guided decision-making (Noonan et al., 2012), in particular in a working memory role when comparing previous to current choices (Wallis and Kennerley, 2011). The ACC in comparison has been implicated in action-outcome learning, choice predictions, prediction errors and surprise signals, more in line with ventral striatum function (Wallis and Kennerley, 2011; Alexander and Brown, 2019). The PCC on the other hand is associated with memory and encoding/retrieval of reward values (for an overview, see Rolls, 2019). Additionally, the insula has been more implicated in the somatosensory experience of rewards, such as taste and touch (Rolls, 2016). This makes it likely that reward sensitivity is different in BED and BN in multiple aspects of reward processing, namely altered reward prediction error, perception of rewards, reward memory, value-guided decision-making, and reward-based learning.

Some care must be taken when interpreting the function of areas, the roles of all aforementioned areas in reward prediction and estimation are still not completely clear. Some seem related to the reward value directly (i.e., ventral striatum), others (i.e., medial OFC, ACC, and insula) mostly to somewhat broader processes like attention elicited by reward (Yan et al., 2016; Uddin et al., 2017; Roefs et al., 2018). The cingulate cortex and insula in particular show activation patterns during presentation of both negative and positive outcomes (Liu et al., 2011). It is of course not necessary that each area has only one specific role. Rather, it is more likely that several loops in this network are involved in the same aspect, for example reward value estimation. Taken together, these results show that differences in this network associated with reward sensitivity in passive viewing tasks are more complex than an unequivocal increase in activity associated with increased processing of reward-related information. It is thus important to investigate the precise function of areas within this network further.

Activity differences in the medial OFC also show up in consummatory tasks (Simon et al., 2016) both in BED and BN, which is consistent with studies in HC (Liu et al., 2011). Activation differences in the dorsal striatum, angular gyrus, and anterior medial PFC seem unique to consummatory tasks (Wang et al., 2011; Filbey et al., 2012; Simon et al., 2016). The dorsal striatum has been mostly associated with learning from rewards, including in instrumental conditioning (O’Doherty et al., 2004; Chen et al., 2015; Tricomi and Lempert, 2015). It is important to note that differences in dorsal striatum activation were only found in a study comparing the effects of methylphenidate with placebo on reward sensitivity, and in a study without a matched control group (Wang et al., 2011; Filbey et al., 2012). The absence of a control group makes determining any differences with HC difficult. Similar to the dorsal striatum, parts of the medial PFC have been implicated in reward learning and implementing behavioral strategies (Alexander and Brown, 2011; Badre and Nee, 2018). This strengthens the case for altered reward learning in BN and BED. Although the angular gyrus shows up in both BN and BED, it is unlikely that it is specific to reward sensitivity. The angular gyrus is a multimodal area that shows activation in a large variety of tasks, and integrates input from across the brain (e.g., from the cingulate cortex, precuneus, insula, PFC; Seghier, 2013). Difference in activation in this area is thus more likely to be a result of difference in activation in other areas in the reward sensitivity network. Lastly, BN patients show in general an overall decrease in activity, while BED patients mostly show an increase in activity compared with HC. Differences in activity direction (negative/positive) in BN and BED could very well be related to a different way of processing received rewards, but not the anticipation of rewards. One possible difference between BN and BED is that the insular cortex has also been associated with body image distortion in BN (Mohr et al., 2011; Yan et al., 2016). Care must however be taken when making inferences, since brain activity does not map onto behavior or experiences directly. Nevertheless, a difference in overall activity in this network is promising in possibly explaining differences between the two disorders.

Regarding the findings of volumetric and connectivity studies, in the current review they show considerable overlap with the reward sensitivity networks. To summarize, in BN, GM in the left insula, left OFC, and ventral PCC is larger in volume, while right OFC, ventral striatum, and dorsal PCC show smaller volumes (Amianto et al., 2013a; Frank et al., 2013; Coutinho et al., 2015; Berner et al., 2018; Westwater et al., 2018). This suggests that reward processing in the OFC might be somewhat lateralized and that the PCC has two separately implicated subregions. Functional connectivity similarly shows increased connectivity between the insula and PFC/OFC (Frank et al., 2016), the ACC and OFC (Lee et al., 2014), and between the caudate nucleus and putamen (Wang et al., 2020). In BED, left ventral striatum volume was also reduced, while ACC volume was found to be increased, and OFC volume seems ambiguously affected (Shafer et al., 2010; Voon et al., 2015). No related connectivity differences were found, although connectivity in the medial dorsal PCC was shown to be increased in BED compared with BN. Indeed, the dorsal PCC, but not ventral PCC, also showed a decrease in GM in BN (Berner et al., 2018). Together with a positive difference in PCC activity in BED during consummatory tasks, this indicates that the PCC could play a different role in reward encoding or attentional processes in BED. Further research into the specific contexts reward sensitivity might be altered in, will help in determining its exact role in BN/BED.

### Cognitive control

In cognitive control tasks, both BN and BED patients show a reduction in activity in frontal areas compared with HC, including the IFG, OFC, dlPFC, superior frontal gyrus, and medial PFC (Marsh et al., 2011; Balodis et al., 2013b; Hege et al., 2015; Voon et al., 2015; Neveu et al., 2018). These are to be expected, as these areas play a large role in controlling our actions (for an overview, see Badre and Nee, 2018). In BED and BN patients, activity differences can be observed throughout the anterior, rostral mid-lateral, and caudal frontal cortex during cognitive control paradigms, including during food-related tasks. Altered processing of food-based decisions is thus likely not limited to decision-making, but perception and sensory feedback of food as well. This could also explain activity differences in the occipital gyrus in BED (Balodis et al., 2013b) through top-down processing.

The caudate nucleus and putamen show activity differences in BED and BN, respectively (Marsh et al., 2011; Voon et al., 2015). The caudate is thought to be crucial for goal-directed control (de Wit et al., 2012; Gasbarri et al., 2014; Watson et al., 2018), and decreased activity along with volumetric and connectivity differences within these areas could explain why a general increase in habitual responding in BED was found (Voon et al., 2015). This has been hypothesized to play a role in the loss of control during binge eating episodes (Voon et al., 2015). The dorsal putamen is shown to be decreased in activity in BN (Marsh et al., 2011) and receives projections from the dorsal posterior insula (Postuma and Dagher, 2006). Activity in medium spiny neurons in the putamen appear to be critical for instrumental learning in primates (Laquitaine et al., 2013). Different parts of the putamen (the posterolateral in humans and caudo-ventral parts in rats) have been associated with habitual responding and habit learning (Balleine and O’Doherty, 2010; de Wit et al., 2012). Reductions in activity during cognitive control tasks could therefore indicate impaired instrumental learning and integration of reward value rather than diminished cognitive control per se. A larger volume of the left putamen in BN may suggest a potential increase in habitual control. Increased functional connectivity of the putamen to subcortical areas, but decreased connectivity with frontal areas (Wang et al., 2020), supports the hypothesis of impaired instrumental learning deficits in BN. However, more studies are needed to confirm whether differences are unique to BN and BED, not only for the striatum but for other areas as well.

Overall, cognitive control is impaired in both BN and BED. Particularly in BN this is illustrated by a reduction of activity in frontal areas such as the OFC, IFG, medial PFC, medial, and superior frontal gyri and is potentially associated with diminished GM volume in these brain areas. The impaired performance is possibly reflective of a decreased influence of the goal-directed network and increased influence of networks associated with habitual responding, potentially creating a bias toward seeking unhealthy eating behaviors.

### Negative affect

Stress-related negative affect has been shown to influence reward sensitivity through activation differences in several areas (Lyu and Jackson, 2016; Collins et al., 2017; Fischer et al., 2017; Wonderlich et al., 2018). BED and BN differ in IFG activation while insula activity differences are unique to BED (Lyu and Jackson, 2016), and right ACC, paracingulate gyrus, precuneus, vmPFC and IFG activity differences are unique to BN (Balodis et al., 2014; Collins et al., 2017; Fischer et al., 2017; Wonderlich et al., 2018). However, since the results in BED are only based on a single study, it is currently not warranted to conclude that BN and BED differ completely as more data are needed to assess the existence of potentially unique activation differences. Activity differences in the ACC, insula, precuneus, and vmPFC have been extensively implicated in similar reward sensitivity and cognitive control tasks outside of acute stress manipulation (Schienle et al., 2009; Simon et al., 2016; Neveu et al., 2018). It is likely that these are not unique to stress-related negative affect, although they could be a result of it.

Differences in activation of the hippocampus in BED and anterior vermis of the cerebellum in BN seem unique to these paradigms that measure reward sensitivity under acute stress. Differences in activation of the anterior vermis are somewhat difficult to evaluate since they have not been typically associated with acute stress or reward processing. As mentioned previously, differences in the amygdala and hippocampal projections to areas associated with reward sensitivity (Salzman and Fusi, 2010; Abivardi and Bach, 2017) can also be observed through structural and functional connectivity studies in BN/BED. BN subjects showed reduced connectivity between the CeA and the hypothalamus in the right hemisphere, and the ventral anterior insula in the left hemisphere (Frank et al., 2016). Connectivity between the CeA and insula has been associated with feeding behavior in rats (Zhang-Molina et al., 2020), with activation of this pathway suppressing food intake. Projections of the CeA and insula also converge in the parasubthalamic nucleus, which together with the CeA and insula modulate feeding behavior of palatable food (Barbier et al., 2020). Through these connections, the insula suppresses activity in the CeA associated with satiety and plays an important role in overfeeding behavior (Stern et al., 2021; for a comprehensive overview of the connections, see Barbier et al., 2020). Aside from the CeA, BN subjects also showed reduced connectivity in the right hemisphere between the BLA and the dorsal anterior insula (dAI), and ventral striatum. It has been shown that the BLA-dAI connections are crucial for reward learning through memory (Gil-Lievana et al., 2020). In particular, the BLA in rats is specifically involved in encoding changes in outcome value, but not the hedonic experience gained from rewards (Parkes and Balleine, 2013; for an overview, see Wassum and Izquierdo, 2015). Crucially, lesions in rat BLA disrupted R-O learning (Balleine et al., 2003), and primate BLA neurons show similar involvement by future-oriented activity to gain rewards (Hernádi et al., 2015). Disrupted BLA-dAI structural connectivity could leave BN (and possibly BED) subjects more reliant on S-R learning mechanisms. Indeed, a similar case can be made for the BLA-ventral striatum connections. Connectivity between the BLA and core of the nucleus accumbens was also necessary for R-O learning in rats (Shiflett and Balleine, 2010), although it is facilitated by a reduction of dopamine release (Jones et al., 2010). Compared with the reward history function of BLA-dAI connectivity, BLA-ventral striatum connectivity is thus more reliant on the current hedonic value of the reward (Wassum and Izquierdo, 2015). Incidentally, BLA connectivity to the lateral and medial OFC have also been shown to be necessary in reward encoding (Malvaez et al., 2019), however this has not yet been shown to be affected in BN/BED.

It is important to mention that interpretations of these studies on stress and reward sensitivity are complicated by not including or reporting cortisol values. One disadvantage of this is that it makes responder classification (Miller et al., 2013) impossible, as this is shown to be a boundary condition in a number of tasks, in particular for the shift from goal-directed to habitual control (Smeets et al., 2019). Indeed, responder classification is also an important factor for food intake in healthy participants. High cortisol reactivity after acute stress exposure was tied to greater food consumption (Epel et al., 2001). It would be interesting to test whether there is an association between cortisol reactivity in BN/BED, and the amount of food intake and the above-described hippocampus activation and amygdala connectivity.

Taken together, these results show that the HPA axis and reward networks are closely connected and differentially affected in BN/BED. Hippocampal activity, which has an inhibitory function on the HPA axis, is affected, and both the CeA and BLA likely play crucial roles in feeding behavior and reward learning. Specifically, this is likely associated with disrupted R-O learning habitual control, which has been shown in humans with BED directly (Voon et al., 2015). This fits well with research showing that after acute stress, healthy participants show a shift from goal-directed to habitual responding in instrumental learning tasks (Schwabe and Wolf, 2009; Wirz et al., 2018; Quaedflieg et al., 2019; Smeets et al., 2019; Hartogsveld et al., 2020). Stress could therefore make BN/BED subjects more likely to rely on S-R associations, while impairing reward values and reducing cognitive control, connecting the three cognitive constructs.

### Future directions

It is crucial to use a multimodal approach that will lead to a better understanding of BN/BED. Such an approach could compromise assessing neural activation during the task itself and combine volumetric and connectivity approaches, which could then establish associations between affected brain areas/connectivity and cognitive processes and ultimately brain network functioning and maladaptive behavioral output. Based on the present review, the three major cognitive constructs affected in BN and BED provide key elements in this approach. There is a number of promising ways that future research could pursue to more precisely delineate the association between brain function and these cognitive constructs. To model mechanisms underlying binges more accurately, future studies could integrate more than just a single of these three cognitive constructs in their paradigm to examine the potential interactive influences they may have. Pavlovian-to-instrumental-transfer (Cartoni et al., 2016; Watson and de Wit, 2018; Mahlberg et al., 2021) is a good example of a paradigm that combines reward sensitivity and cognitive control. Indeed, this review shows that the networks involved in Pavlovian conditioning show considerable overlap with those responsible for the transfer to instrumental learning and reward sensitivity (Corbit and Balleine, 2016). Another promising approach is to investigate how stress influences the balance between brain networks involved in instrumental learning in BN/BED, as stress could make subjects more likely to rely on stimulus-response associations (model-free) while also impairing reward value attribution to an outcome. It is likely that the balance between goal-directed (model-based) and habitual (model-free) behavior is affected in BN/BED and associated with structural brain differences, but unfortunately there is only one study (Voon et al., 2015) investigating this in patients. However, Voon et al. (2015) only investigated volumetric GM associations of a subsample of all the subjects performing the instrumental learning task. To establish a more complete understanding of these disorders, associations between GM volume, brain activation, structural and functional connectivity as well as impaired cognitive functioning needs to be determined in larger samples. Advanced designs and analyses such as multivariate pattern analysis for both activity and connectivity (Anzellotti and Coutanche, 2018) and algorithmic network approaches (Bielczyk et al., 2019) show considerable promise and could be very effective in providing unambiguous results.

BN and BED seem to affect men and women equally (Hudson et al., 2007; Guerdjikova et al., 2017). Thus, future research also has to specifically address the lack of research on men with BN and BED, as most studies discussed in this review only involved women. This may be problematic, since there appears to be sexual dimorphism of the mesocorticolimbic system, particularly involving stress responses (Gillies et al., 2014; Douma and de Kloet, 2020). Moreover, sex hormones influence glucocorticoid secretion and functioning of ventrotegmental dopamine neurons. The ventrotegmental area is in turn connected to the ventral striatum and PFC, which makes this network very likely to play a central role in reward sensitivity, particularly after acute stress exposure. This potentially could increase reward values for certain foods and the likelihood of binge eating. It has also been shown that the effects of acute stress are different for men and women, both when influencing decision-making and the way it is involved in certain psychiatric disorders (Bangasser and Valentino, 2014; Bale and Epperson, 2015; Georgiou et al., 2018; Wellman et al., 2018). Therefore, stress may differentially affect decision-making and binge eating in men and women with BN and BED. More refined research is needed to confirm these findings in male samples to identify their exact role in BN and BED.

Taken together, we show in this review that BN and BED are characterized by differences in three networks, involving the prefrontal and cingulate cortex, insula, striatum, amygdala and hippocampus. Each of these networks is associated with different cognitive constructs, namely reward sensitivity, cognitive control, and negative affect. Numerous key nodes in these networks are also different in volume and connectivity compared with HC. Moreover, some of the studies discussed indicate that alterations in volume are present in current, but not in recovered, patients, and that these alterations correlate positively with symptom severity (Wagner et al., 2006; Cyr et al., 2017). Nevertheless, the number of studies on BN and BED is still limited. Therefore, caution must be taken when drawing strong conclusions about these disorders based on the available evidence. A related issue is caused by the relatively small samples in the studies with BN/BED patients (although studies investigating subclinical subjects have included larger samples). Especially in smaller samples, smaller differences that may be present might not be substantial enough to be detected because of a lack of statistical power. This leaves the possibility of smaller, but relevant structural or functional differences in brain organization still undiscovered in BN and BED. There are some differences found in volumetric and connectivity studies that do not map onto the three cognitive constructs proposed in this review. These are the inferior parietal cortex (Marsh et al., 2015; Westwater et al., 2018; Wallace et al., 2020), middle/inferior temporal gyrus (Marsh et al., 2015; Westwater et al., 2018), paracentral lobule (Amianto et al., 2013a), lingual gyrus (Marsh et al., 2015; Stopyra et al., 2019), and the fusiform gyrus (Marsh et al., 2015). These areas might be associated with other constructs not discussed here (e.g., body image distortion, other psychiatric comorbidities such as depression or anxiety disorder), or they might not map onto any construct at all. In addition, most of these differences are only confirmed by a limited number of studies with relatively small sample sizes. It is therefore essential that more high-powered multimodal studies are performed to improve our understanding of the brain-behavior connection in BN/BED.

#### Search strategy and selection criteria

A literature search was performed for this narrative review in PubMed and Google Scholar. We used the following search terms: ((bulimia nervosa) OR (binge eating disorder)) AND ((volumetric) OR (cortical thickness) OR (diffusion) OR (resting state)) OR (((reward sensitivity) OR (cognitive control) OR (impulsivity) OR (decision-making) OR (instrumental learning) OR (negative affect) OR (stress) OR (cortisol) OR (HPA axis) AND (fMRI)). The reference lists of the included articles were also searched for additional potentially relevant articles. The final search was conducted on the 10^th^ of November 2020, yielding a total of 463 articles. Our exclusion criteria for this narrative research were: (1) not including participants that are either BED or BN patients or experienced BED or BN symptoms; (2) not including measures of brain structure, connectivity, or activity during relevant tasks; (3) the article scores poor on our quality assessment (see below) and/or includes extremely small samples; (4) review articles not reporting original data; (5) conference proceedings without full report publication. After removing excluded articles and duplicates, the final number of eligible studies was 45. Results were extracted from both the main paper and supplementary materials available online, and all tests are reported in Tables 1, 2.

#### Quality assessment

**Figure 5.**
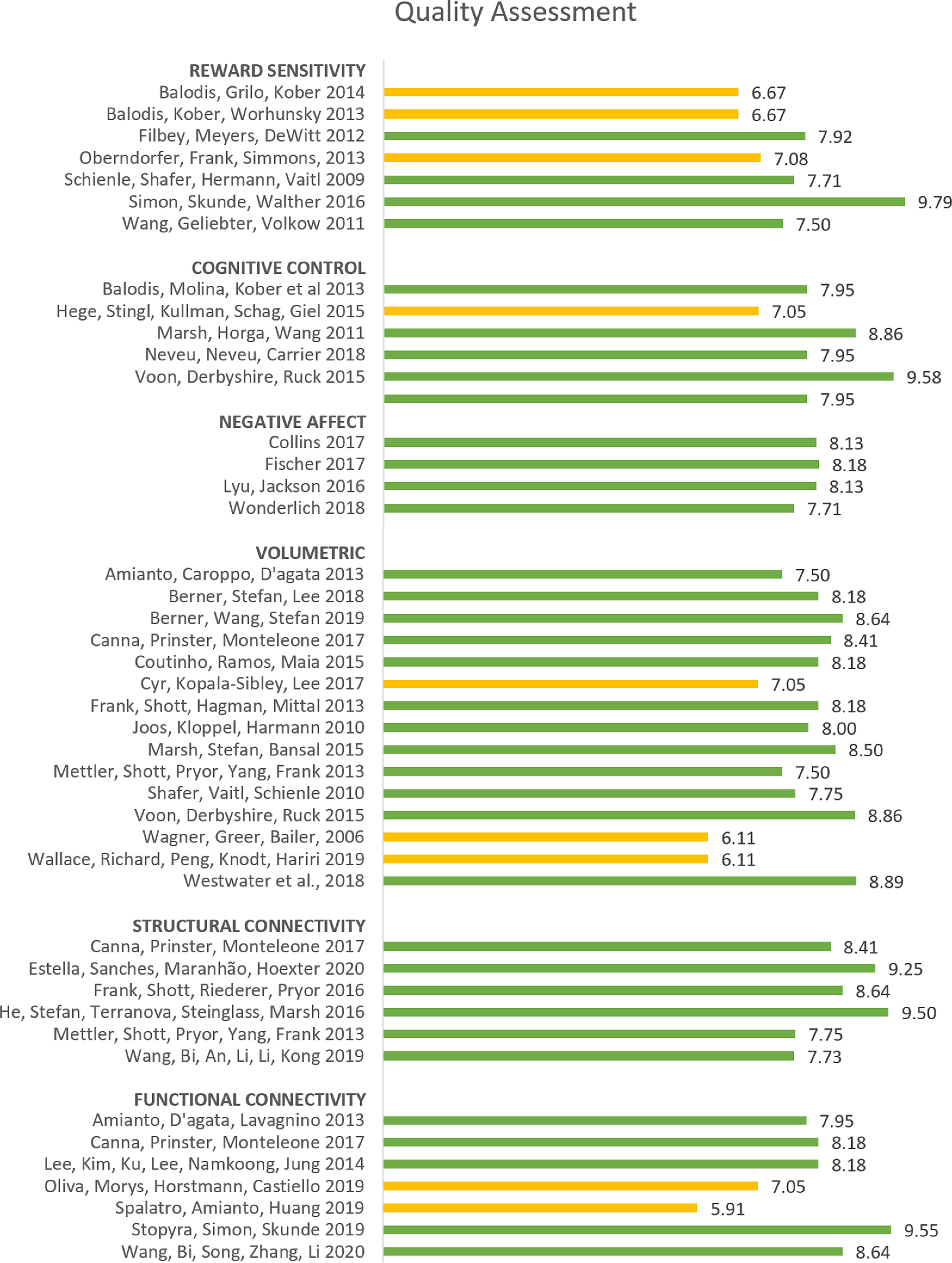
Summary of quality assessment performed for all included MR studies (related to the three cognitive constructs, volumetric, or connectivity). The studies were evaluated by two independent raters, based on 12 criteria (see Concluding Remarks, Quality assessment). Each criterion was scored with 1 point (+), 0.5 point (±), or 0 points (–). The corrected average score [(total points/number of applicable criteria × 10)/2] is presented in this figure, with yellow indicating a rating of “fair” (<7.5) and green a rating of “good” (≥7.5).

Quality of the studies was evaluated based on the nine criteria described in Wolters et al. (2019): (1) description of participants; (2) description of imaging procedure and instructions; (3) description of psychological task; (4) description of spatial normalization procedure; (5) specification of regions of interest; (6) suitability of imaging pipeline for analyzing imaging data; (7) multiple testing problem; (8) support of all empirical claims by statistical tests; (9) quality of tables and figures. We added three criteria that were particularly relevant in evaluating the quality of studies using BN and BED samples: (10) quality of assessment of BN/BED status (e.g., formal diagnosis, questionnaire); (11) correct matching of control group to patient group; and (12) the size of sample within each subgroup. Each criterion could be scored with 1 point (+), 0.5 points (±), or 0 points (–). Total score was calculated and corrected for number of applicable criteria (total score/number of applicable criteria × 10). A score of 7.5 or higher was considered as good quality, a score between 4 and 7.5 as fair quality, and a score of four or less as poor quality (Wolters et al., 2019). Quality of papers was considered while interpreting and discussing the results of the data synthesis. Quality assessment was performed independently by two researchers. There were no major discrepancies in overall quality rating between the assessors (poor, fair, good). Differences between the assessors for individual criteria (e.g., 9. quality of tables and figures) also did not exceed 0.5 points. The average score between the two researchers is reported in Figure 5.

## Synthesis

Reviewing Editor: Aniko Korosi, Universiteit van Amsterdam Decisions are customarily a result of the Reviewing Editor and the peer reviewers coming together and discussing their recommendations until a consensus is reached. When revisions are invited, a fact-based synthesis statement explaining their decision and outlining what is needed to prepare a revision will be listed below. The following reviewer(s) agreed to reveal their identity: Marie Barbier.

Reviewer 1

The manuscript provides advances in the field as it in principle offers a review on the 3 core neuropsychological deficits seen in binge eating EDs (BED & BN), and on their neurobiological correlates.

There is a relative lacune on this topic in the literature, so the review could fill an important gap.

This review sets out to outline the presumably three neuropsychological domains thought to be disturbed in eating disorders characterized by binge eating (Binge Eating Disorder [BED] and Bulimia Nervosa [BN]). The three proposed domains of importance are:

1. Reward processes

2. Cognitive control / impulsivity

3. Stress, negative affect, negative urgency

The authors then proceed to discuss human imaging evidence for affected neural circuits linked to those 3 domains in context of BN/BED, and follow up with a discussion of more structural problems in brains of BED/BN patients.

I appreciate that the authors have tried to parse their review along the lines of 3 cognitive domains that likely contribute much to the disorder. The review in principle addresses an important gap in the literature in this regard.

My main points are as follows:

1. The structure of the ms invites redundancy and some ambiguity on where specific information will be provided. As just one example, relevant information on how cognitive control processes are perturbed in BN/BED are divided over p5-6 (as a sort of quite elaborate intro on this topic). Then there is another topic. And then again this point gets revisited on p8-9.

The same formula is in principle used for all three domains, though there are also clear differences in how much each of them is immediately focused on explaining general concepts vs mentioning specific differences in BED/BN. For instance, the p4 section on Reward sensitivity deals with very broad conceptualizations of reward (mainly related to reward learning rather than reward sensitivity actually), and hardly puts it into context of BED/BN (just a few lines at the end, L117-120), whereas the aforementioned equivalent section on Cognitive control is already heavily on occurrence of such issues in specifically BED/BN patients.

Those elements together make the review feel a bit disjointed at times. It would seem to me that a cleaner division between sections on “behavioral evidence that specific construct X is perturbed in BN/BED” eventually followed by just a section purely on “neural circuit perturbations linked to construct X” (where I also feel that integrating rather than separating the structural and functional evidence for involvement of brain regions could make sense), would make it easier to read.

2. The delineation of the neural circuits of interest is not so clear. Rather than attempting to a priori define which brain regions are known to be crucial to evaluate in terms of Reward Sensitivity (e.g. (Ventral) Striatum / OFC / midbrain) and then following up with what is known about dysfunction of such regions in context of BED/BN, another route is often taken. Namely, listing of brain regions that showed alterations in BED/BN patients in tasks with, e.g., reward or control or stress components. The final outcome of such a domain is also rather difficult to interpret. I recognize that this may also be due to relative lack of available data though. Indeed, usually cited study numbers for a given region end up being rather low. Also the benefit of grouping together BED and BN also clearly has its limitations, as for instance the reward sensitivity domain findings are very inconsistent across those diseases for given brain regions. There are usually very few studies that can be cited for a given brain region. Some of that interpretation of what a brain region might do only comes at the very end (Sections 4). It may also here prove to be more legible if some of that explanation on the role of regions that are being named, was clarified earlier on.

3. Linked to the point above. The Figures currently only permit describing increased vs decreased activity of a (small) set of regions. There is no incorporation in these figures of a ‘no difference found’ option. I for one would find it more revealing to know how many studies that were assessed by the authors didn’t find activity differences in known key reward regions (e.g ventral striatum) (or stress regions like amygdala) of BED/BN patients, rather than knowing only which regions (often with much less canonical involvement in the construct) did show effects.

4. Choice of axes length in the figures seems a bit arbitrary. There are sections where the number of study doesn’t exceed 1 for any region, where the axis nonetheless runs until 4 studies (e.g. Fig 3, BN stress) and others where similarly the number of studies doesn’t exceed 1, where the axis only runs until 1 (e.g. Fig 3, BED stress). Some more consistency on this front would be good, to also at a glance see even more clearly that there is great paucity of data on many regions for these constructs.

Overall it’s an interesting review on a topic with clear gaps in knowledge. The ms seeks to parse its content along clear divides of cognitive domains and their circuits, succeeds in part, but also has too much blending of such lines and fragmentation/redundancy in the text for optimal legibility to my taste. Improvements on that front would make it a valuable review though.

Reviewer 2

I would like to thanks the authors for this work, the paper is well written and detailed. The manuscript will provide advances in the field because it’s the first time that authors show three cognitive contructs (reward sensitivity, cognitive control and negative affect) underlying binge eating disorder and bulimia nervosa explained in brain activity differences (volumetric differences, structural and functional connectivity). They also focused on the frontal/cortical brain areas functions and provide suggestions for future research in this field.

The authors describe and compare two pathologies, the bulimia nervosa and the binge eating disorders, based on 45 studies (according to their exclusion criteria).

Each parts of their work is followed by a summary figure that helps to check quickly the difference or the similarities between the diseases.

However, several points can be improved or clarified:

1. Line 232, please add the abbreviation of BMI?

2. Line 395: “only one study...”, please cite the study in question.

3. Line 405: “Several studies...”, please cite some of them.

4. Line 505: I think there is a mistake in the word “mesocorcicolimbic” (mesocorticolimbic).

5. The authors describe the negative affect contruct and associate it with the stress and so with the HPA axis, but can we say about the role of other peptides or hormones such as the CRF (corticotropin-releasing hormone, ghrelin..., and their impact on the other contructs as on the reward process?

6. What about the posterior structures in the brain, especially about the hypothalamus (lateral areas and periventricular nuclei) and the ventral tegmental area. It is well known that this last one is important in the dopamine release in the “reward network” as the accumbens nucleus and the prefrontal cortex. As consequence these structures could be involved and show difference in their activity in BN and BED.

7. Lines 756 to 760: The authors speak about hypothalamus, CEA and insular cortex. About the CEA and insula, there is an other paper recently published (Stern et al., 2021, Cell Metabolism33, 1-15June 1, 2021) that is interesting in term of food intake modulation.

Moreover, concerning the CEA and hypothalamic connections there is an interesting paper (Proc Natl Acad Sci U S A. 2020 Jul 7;117(27):15967-15976. doi: 10.1073/pnas.2004914117) in which the authors describe a cortico-amygdala-hypothalamic network involving a nucleus in the posterior lateral hypothalamus, the parasubthalalic nucleus (PSTN), associates in neophobia and sickness states.

I cite: “the PSTN complex modulates behavior in response to hedonic factors normally inducing a “reward effect.” The PSTN complex is involved in a “non-feed” response equivalent of a “non-reward” evaluation: “I don’t want to consume this food, recognized as good, because I am not in a conducive emotional or physiological state.”
